# Nosocomial Respiratory Syncytial Virus in the Post-Pandemic Era: Evolving Epidemiology and Integrated Prevention Strategies

**DOI:** 10.3390/healthcare14193335

**Published:** 2026-10-06

**Authors:** Po-Hsiu Huang, Pei-Hsuan Huang, Po-Yu Liu, Ting-Kuang Yeh

**Affiliations:** 1 Division of Infectious Diseases, Department of Internal Medicine, Taichung Veterans General Hospital, 1650 Taiwan Boulevard, Section 4, Taichung 407219, Taiwan; 2 Infection Control Center, Taichung Veterans General Hospital, 1650 Taiwan Boulevard, Section 4, Taichung 407219, Taiwan; 3 Genomic Center for Infectious Diseases, Taichung Veterans General Hospital, 1650 Taiwan Boulevard, Section 4, Taichung 407219, Taiwan; 4 School of Medicine, National Yang Ming Chiao Tung University, No. 155, Section 2, Linong Street, Beitou District, Taipei 112304, Taiwan; 5 Department of Post-Baccalaureate Medicine, College of Medicine, National Chung Hsing University, 145 Xingda Road, South District, Taichung 402202, Taiwan; 6 Doctoral Program in Translational Medicine, Rong Hsing Translational Medicine Research Center, National Chung Hsing University, 145 Xingda Road, South District, Taichung 402202, Taiwan; 7 Institute of Clinical Medicine, National Yang Ming Chiao Tung University, No. 155, Section 2, Linong Street, Beitou District, Taipei 112304, Taiwan

**Keywords:** respiratory syncytial virus, nosocomial infection, healthcare-associated infection, infection prevention and control, seasonality, immunoprophylaxis, healthcare workers

## Abstract

**Highlights:**

**What are the main findings?**

- Post-pandemic RSV activity has been earlier and less predictable than before 2020, and periods of concurrent circulation with influenza and SARS-CoV-2 have been observed, undermining calendar-based hospital preparedness.
- Healthcare-associated RSV disproportionately affects neonates, immunocompromised patients, and frail older adults, prolonging hospital stays and increasing direct costs, and asymptomatic or mildly symptomatic healthcare workers are one potential source of introduction and onward transmission, alongside patients and visitors.

**What are the implications of the main findings?**

- Hospital infection-prevention programs should be triggered by real-time surveillance rather than fixed seasons and layered across hand hygiene, transmission-based precautions, environmental disinfection, rapid diagnostics, and supportive workforce policies.
- Long-acting monoclonal antibodies and maternal and older-adult RSV vaccines reduce severe RSV disease in eligible groups; their contribution to hospital infection prevention is indirect, and direct evidence that they prevent healthcare-associated transmission is currently lacking.

**Abstract:**

Background/Objectives: Pandemic non-pharmaceutical interventions virtually eliminated respiratory syncytial virus (RSV) circulation in 2020–2021, and their relaxation was followed by unprecedented off-season resurgences. This narrative review, informed by a structured literature search, synthesizes current evidence on healthcare-associated RSV (HA-RSV), including transmission dynamics, vulnerable inpatient populations, and integrated prevention strategies, in the post-pandemic era. Methods: We searched PubMed/MEDLINE and Europe PMC (January 2000–October 2025) using two complementary strategies, supplemented by WHO and CDC guidance. Primary hospital-based studies reporting laboratory-confirmed HA-RSV cases formed the core evidence set; surveillance reports, systematic reviews, and guidance were used as labeled contextual evidence and synthesized narratively. Results: Twenty-nine primary studies from neonatal, pediatric, adult, geriatric, and immunocompromised settings provided direct evidence on HA-RSV, which accounted for 3–15% of RSV hospitalizations depending on setting and case definition and was associated with prolonged stay, escalation of respiratory support, higher costs, and, in children, higher mortality than community-onset infection. Universal masking and pre-admission screening were followed by reductions in HA-RSV in most before–after studies. Post-pandemic surveillance shows earlier, less predictable RSV activity and periods of concurrent circulation with influenza and SARS-CoV-2. RSV spreads mainly through short-range respiratory particles and contaminated hands and surfaces; asymptomatic or mildly symptomatic healthcare workers are one potential source of introduction. Conclusions: Prevention of HA-RSV rests on hand hygiene, transmission-based precautions, environmental disinfection, rapid molecular diagnostics, and supportive healthcare-worker and visitor policies, escalated according to local surveillance rather than the calendar. Immunoprophylaxis and vaccination reduce severe RSV disease in eligible groups, but direct evidence that they prevent healthcare-associated transmission is lacking.

## 1. Introduction

Respiratory syncytial virus (RSV) is among the most prevalent causes of acute lower respiratory infection across the human lifespan. Among children younger than 5 years, RSV is a leading cause of acute lower respiratory infection and hospitalization worldwide. Among older adults in the United States, RSV causes substantial hospitalization and mortality burdens [1], underscoring the threat at both ends of the age spectrum.

Healthcare-associated RSV (HA-RSV), a term we use throughout as a synonym for “nosocomial” and “hospital-acquired” RSV, as in the source literature, denotes laboratory-confirmed RSV infection with onset after admission that was neither present nor incubating on admission. HA-RSV compounds this burden. Some surveillance systems also use an expanded definition that captures patients transferred from post-acute care or recently discharged; where this review uses such a definition, it states it explicitly. Healthcare facilities concentrate vulnerable patients—premature neonates, immunocompromised children, transplant recipients, and frail older adults—while enabling close contact among patients, visitors, and staff. Outbreak investigations have reported substantial secondary transmission in neonatal and pediatric units [2,3]. HA-RSV can prolong hospitalization and increase direct medical costs [4]. Healthcare workers with mild symptoms may continue working while infected and, together with patients and visitors, can contribute to onward transmission [5,6].

The COVID-19 pandemic upended this paradigm: non-pharmaceutical interventions virtually eliminated RSV circulation in 2020–2021, and their relaxation was followed by unprecedented off-season resurgences with shifted timelines and higher peaks [7,8,9]. An “immunity debt” in unexposed cohorts has been proposed as one explanation for these compressed, severe waves; however, other factors, including the accumulation of RSV-naïve infants across successive birth cohorts, waning maternal and population antibody levels, changes in testing intensity and surveillance, and viral interference, are likely to have contributed, and their relative importance remains uncertain [10,11,12,13,14].

For infection-prevention teams, this erosion of seasonality complicates forecasting, stockpiling of diagnostics and personal protective equipment (PPE), and timely deployment of monoclonal antibodies or vaccines. Hospitals therefore need to shift from a calendar-based stance to year-round vigilance supported by flexible surge plans and rapid diagnostics.

This narrative review, informed by a structured literature search, synthesizes the current understanding of RSV transmission dynamics, vulnerable patient groups, and evidence-based prevention strategies in hospital settings globally, emphasizing how the COVID-19 era has transformed RSV epidemiology and its implications for preparedness, surveillance, and outbreak management. Throughout, we distinguish direct evidence from HA-RSV studies from contextual evidence from general hospitalized or community RSV populations, and we indicate the source and level of evidence supporting each statement.

## 2. Methods

### 2.1. Review Design and Search Strategy

This narrative review draws on a structured, reproducible literature search. Selected elements of systematic-review methodology (predefined search strings in multiple databases, independent dual screening, and standardized data charting) were adopted to limit selection bias. Formal risk-of-bias grading and quantitative synthesis were not performed, contextual sources outside the core study set were included at the authors’ discretion, and no protocol was registered; the review is therefore presented as a narrative review rather than as a systematic or scoping review.

**Figure 1:**
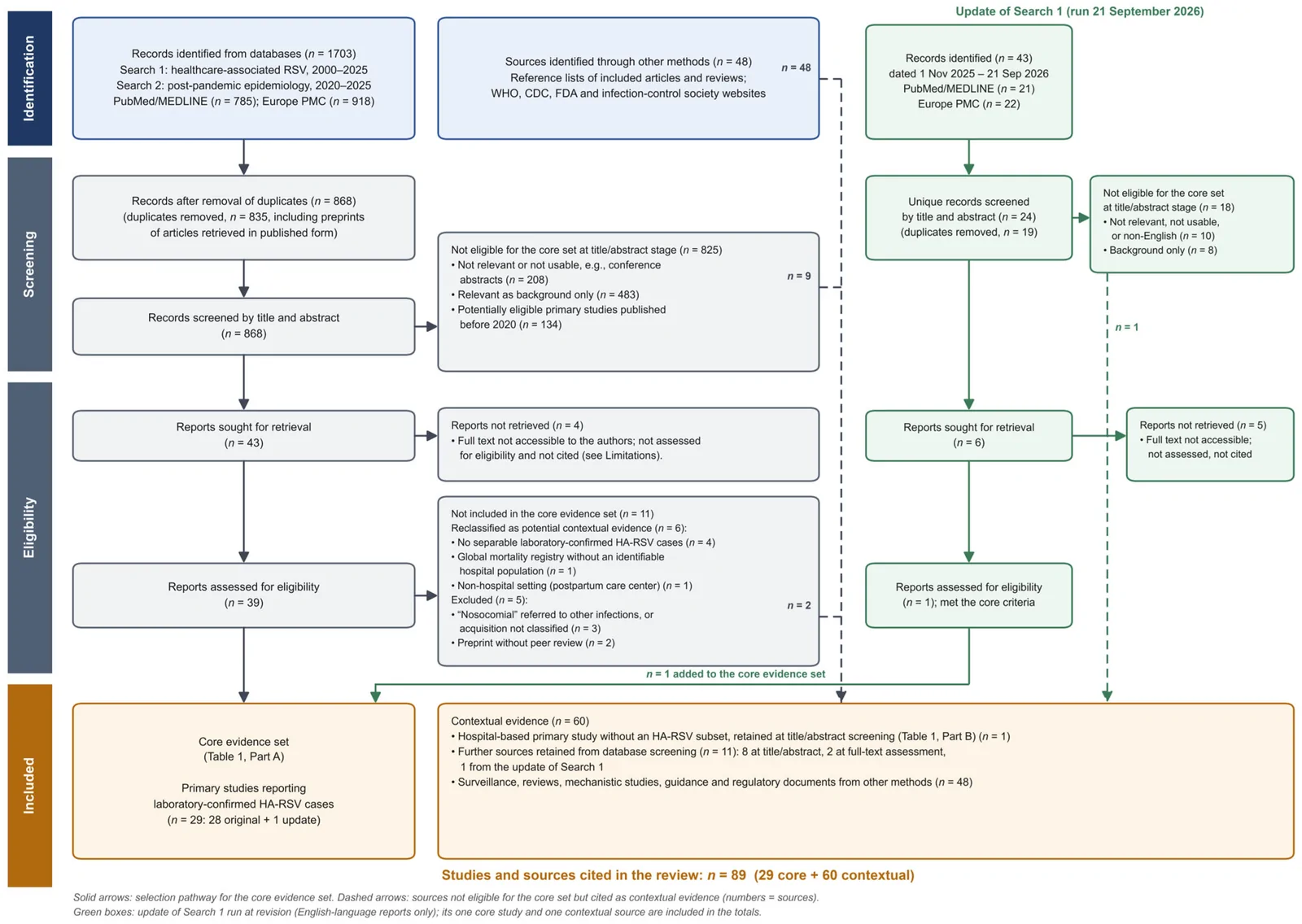
Flow diagram of the structured literature search and study selection, adapted from the PRISMA 2020 template. Records were identified from two electronic databases (PubMed/MEDLINE and Europe PMC; two complementary searches: healthcare-associated RSV without pandemic-related terms, and post-pandemic epidemiology) and from hand-searching of reference lists and guidance websites, deduplicated, screened by title and abstract, and assessed in full text against the eligibility criteria; reports that could not be retrieved are shown as a separate step. The right-hand column shows the update of Search 1 run on 21 September 2026, for records dated after the original cut-off; its one core study and one contextual source are included in the totals. We classified studies as the core evidence set (primary studies reporting laboratory-confirmed healthcare-associated RSV cases; Table 1, Part A) or as contextual evidence (Table 1, Part B, and other sources cited in the text). Numbers refer to records at each stage; reasons for full-text exclusion are listed in the figure. Solid arrows show the selection pathway for the core evidence set; dashed arrows show records and sources that were not eligible for the core set but are cited as contextual evidence, with the number of such sources indicated on each arrow.

Two electronic databases, PubMed/MEDLINE and Europe PMC, the latter additionally indexing PubMed Central, preprint servers, and other sources not covered by MEDLINE, were searched for publications dated 1 January 2000 to 31 October 2025 (searches run on 16 September 2026, and 17 September 2026, respectively). Two complementary strategies were used. Search 1 targeted healthcare-associated RSV and combined RSV terms with terms for nosocomial, hospital-acquired, or healthcare-associated infection and hospital outbreaks, deliberately without pandemic-related terms, so that pre-pandemic evidence on transmission and prevention was captured; the PubMed string was (“Respiratory Syncytial Virus, Human” [Mesh] OR “Respiratory Syncytial Virus Infections” [Mesh] OR “respiratory syncytial virus” [tiab] OR RSV [tiab]) AND (“Cross Infection” [Mesh] OR nosocomial [tiab] OR “hospital-acquired” [tiab] OR “hospital acquired” [tiab] OR “healthcare-associated” [tiab] OR “healthcare associated” [tiab] OR “health care-associated” [tiab] OR “hospital outbreak” [tiab] OR “hospital-onset” [tiab]). Search 2 targeted post-pandemic epidemiology and combined RSV terms with COVID-19/pandemic terms, seasonality or resurgence terms, and hospital terms, restricted to publications from 2020 onward. The complete database-specific strategies are provided in Supplementary Table S1. Search 1 was subsequently updated on 21 September 2026, in both databases for records dated 1 November 2025 to 21 September 2026; for this update, eligibility was restricted to English-language reports at the screening stage, records were otherwise screened in the same way as the original search, and the results are reported separately in Section 2.2 and Figure 1. Search 2 was not updated beyond 31 October 2025. We also searched the reference lists of included articles and pertinent reviews, and searched WHO, CDC, FDA, and infection-control society websites for guidance, regulatory documents, and grey literature. Because recommendations on RSV immunization and isolation precautions change frequently, these websites were last accessed on 16 September 2026 (FDA product information on 20 September 2026), so the cited guidance reflects the most recent versions available. One narrative review of RSV outbreaks in healthcare facilities published after the October 2025 cut-off was identified during this check and is cited as contextual evidence only (Section 3.3).

### 2.2. Eligibility Criteria and Study Selection

Articles were eligible if they involved human subjects and addressed RSV transmission, epidemiology, or infection prevention in acute-care or long-term hospital settings, with extractable data or substantive clinical commentary. We excluded animal studies and studies confined to community-acquired infection without hospital-based data. For the update of Search 1, eligibility was restricted to English-language reports at the title/abstract stage. All records that reached full-text assessment were published in English, so no translation was required.

Two categories of sources were distinguished a priori. The core evidence set comprised primary hospital-based studies (cohort, surveillance, or outbreak investigations conducted in one or more identifiable hospitals or wards, so that cases could be related to the patient population from which they arose) published in peer-reviewed journals from January 2020 through the applicable search cut-off (31 October 2025, for the original search and 21 September 2026, for the update of Search 1) that reported laboratory-confirmed HA-RSV cases in any age group (conference abstracts and preprints were not eligible); these are summarized in Table 1, Part A, and constitute the direct evidence on HA-RSV in this review. The 2020 publication threshold is a pragmatic boundary chosen so that the core set reflects the pandemic transition and current diagnostic and infection-prevention practice; it does not mark the introduction of molecular diagnostics, and earlier primary studies of HA-RSV identified by Search 1 are cited as pre-2020 evidence where they remain the best available (Section 2.3). Contextual evidence comprised (i) hospital-based primary studies from the same period without a reported HA-RSV subset that were retained because they described post-pandemic changes in RSV seasonality or clinical presentation (Table 1, Part B), and (ii) surveillance reports, systematic reviews and meta-analyses, mechanistic studies, earlier outbreak investigations, economic analyses, and guidance documents identified through the searches. Contextual sources were used to describe the epidemiological background, transmission mechanisms, and current prevention guidance, which the core studies do not cover, and are labeled as such in the Results and Discussion; data from general RSV inpatient populations without a separable HA-RSV group were not used to estimate the attributable burden of HA-RSV. Primary studies of HA-RSV published before 2020 and systematic reviews of HA-RSV also provide direct evidence on HA-RSV; where they are used for this purpose, they are identified as pre-2020 or secondary evidence.

Two reviewers (P.-H.H. [Po-Hsiu Huang] and T.-K.Y.) screened titles and abstracts independently, resolved discrepancies by consensus or with a third reviewer (P.-Y.L.), and then assessed full texts against the eligibility criteria. In total, 1703 records were identified (PubMed/MEDLINE, n = 785; Europe PMC, n = 918; counts summed across Searches 1 and 2). After removal of 835 duplicates (including preprints of articles already retrieved in published form), 868 records were screened, of which 825 were not eligible for the core evidence set at the title/abstract stage (208 not relevant or not usable, such as conference abstracts; 483 relevant as background only; and 134 potentially eligible primary studies published before 2020). We sought full text for all 43 records. We could not retrieve four reports because the full text was not accessible to the authors, so we did not assess them for eligibility. Of the 39 reports assessed, 28 primary studies met the criteria for the core evidence set, 6 were reclassified as potential contextual evidence (4 without separable laboratory-confirmed HA-RSV cases, 1 global registry of RSV-related deaths without an identifiable hospital population, and 1 conducted in a non-hospital postpartum care center), and 5 were excluded (3 in which “nosocomial” referred to other infections or RSV acquisition was not classified, and 2 preprints without peer review). We assessed all 28 studies in the core evidence set on full-text review. In addition to the core studies and one hospital-based study retained as contextual primary evidence (Table 1, Part B), we cited 11 additional sources identified through the database searches (including 1 from the update search) and 48 sources identified through hand-searching of reference lists and guidance websites as contextual evidence. The update of Search 1 identified 43 records (PubMed/MEDLINE, n = 21; Europe PMC, n = 22), of which 24 were unique; 18 were not eligible for the core set at the title/abstract stage (10 not relevant, not usable, or non-English; 8 relevant as background only) and 6 were sought in full text. Five of these could not be retrieved and were not assessed; the one report assessed met the core criteria and was added to the core evidence set (Section 3.1). One further record from the update is cited as contextual evidence (Section 3.3). The search and selection process, including the update, is summarized in Figure 1.

### 2.3. Data Charting, Consideration of Study Quality, and Synthesis

For each study in the core set, the design, setting, population, study period, case definition of HA-RSV (including any temporal criterion after admission), diagnostic method, number of HA-RSV cases relative to the reported denominator, infection prevention and control (IPC) measures, principal outcomes, main quantitative findings, and major limitations were charted into a standardized spreadsheet by one reviewer and verified by a second; these characteristics are displayed in Table 1. We did not undertake a formal risk-of-bias assessment and assigned no certainty ratings. Instead, the methodological limitations of each core study (design, sample size, case definition, diagnostic method, and setting) were recorded in Table 1 and taken into account when interpreting the findings: single-center retrospective series and outbreak reports were interpreted more cautiously than multicenter cohorts or population-based surveillance, and the type of source (primary study, systematic review, or guidance) is stated wherever a statement rests on it. The absence of formal appraisal limits the conclusions that can be drawn about the certainty of the evidence. The working definition of HA-RSV adopted for this review was laboratory-confirmed RSV infection with symptom onset or first positive test after the incubation period following admission (typically ≥3–5 days), or membership of a molecularly confirmed in-hospital cluster; because the temporal criterion varied across studies (typically from 48 h to 5 days), the definition used by each study is reported rather than harmonized. We synthesized data narratively around five themes: epidemiological trends, transmission mechanisms, high-risk populations, infection-prevention interventions, and post-COVID-19 shifts. We explored divergent findings qualitatively by ward type, diagnostic method, and regional seasonality. Where a statement rests on formal guidance, on a systematic review or meta-analysis, or on a single primary study, this is indicated in the text.

## 3. Results

### 3.1. Direct Evidence on Healthcare-Associated RSV: Core Studies

Twenty-nine primary studies published between 2020 and 2026, with data-collection periods spanning 2005–2023 and conducted in 16 countries or territories, reported laboratory-confirmed HA-RSV cases in hospitalized patients and form the core evidence set (Table 1, Part A). Classified by principal design, with each study counted once, they comprise five outbreak investigations [6,15,16,17,18] (three NICUs, a long-term geriatric hospital, and an adult hematology ward), four of which used molecular typing or whole-genome sequencing and one of which [16] did not; eleven single- or multicenter cohort, surveillance, or cross-sectional studies [19,20,21,22,23,24,25,26,27,28,29]; three matched-cohort studies of HA-RSV outcomes [4,30,31]; seven evaluations of infection-prevention measures [32,33,34,35,36,37,38], comprising four before–after comparisons [32,34,36,38], one interrupted time-series [35], one non-randomized parallel comparison [33], and one single-arm surveillance study [37]; a single-season prospective study of nosocomial versus community-acquired RSV in a mixed adult–pediatric hospital [39]; one analysis of population-based surveillance [40]; and one retrospective whole-genome-sequencing surveillance study of healthcare-associated respiratory viruses in three hospitals that identified RSV transmission clusters [41]. Diagnostic methods included PCR-based assays, sometimes supplemented by rapid antigen testing; one long-running study (2005–2020) also used viral culture in its earlier years [21]. The temporal criterion defining healthcare-associated acquisition ranged from ≥48 h to ≥5 days after admission (≥8 days for “definite” cases in one study) or was based on the virus-specific incubation period, and five studies did not state an explicit criterion; denominators also varied (matched controls, all RSV-positive admissions, all hospitalized RSV-LRTI, patient-days, admissions, or population), so that incidence estimates and proportions are not directly comparable across studies. We retained a single hospital-based study from Thailand that did not report a nosocomial subset only as contextual evidence on post-pandemic seasonality and clinical presentation (Table 1, Part B).

**Table 1:** Characteristics of primary studies included in this review. Part A: studies reporting laboratory-confirmed healthcare-associated RSV cases (core evidence set), grouped by setting. Part B: hospital-based study without a reported healthcare-associated subset, retained as contextual evidence.

Study [Ref.], Country	Design; Setting; Study Period	HA-RSV Definition (Temporal Criterion)	HA-RSV Cases/Denominator	Diagnostic Method	IPC Measures Reported	Principal Outcome; Main Quantitative Finding	Major Limitation
Part A. Studies reporting laboratory-confirmed healthcare-associated RSV cases in hospitalized patients (core evidence set, *n* = 29) A1. Neonatal and pediatric settings							
Comas-García et al. [4], Mexico	Retrospective matched cohort; NICU of a public general hospital; July 2012–December 2017	Not explicitly stated (nosocomial RSV detected by RT-PCR during NICU stay; median age at detection 16.5 days)	24 HA-RSV infants vs. 24 controls matched for gestational age, birth weight and period of hospitalization; NICU denominator not reported	RT-PCR	Not reported	After RSV detection: median 24 vs. 13 days to discharge (total stay 44 vs. 35.5 days); antibiotics 45.8% vs. 8.3%; further nosocomial infection 45.8% vs. 0%; median direct cost US $3587 vs. US $1124; total charges 41.9% higher (≈US $1836 per infant); 1 death (4.2%) vs. 0	Single center; small n; no incidence denominator; cases more often ventilated before RSV acquisition (58.3% vs. 16.7%)
Silva et al. [15], Brazil	Prospective outbreak investigation with genotyping; 28-bed NICU; May–August 2013	Not explicitly stated (all cases NICU inpatients since birth, identified by twice-weekly universal screening)	Two consecutive outbreaks: 10/17 infants (attack rate 58.8%, genotype ON-1) and 14/22 (63.6%, genotype NA-2); 24 infected infants in total	Real-time RT-PCR; G-gene sequencing	Contact and droplet precautions; three-cohort segregation of infants and staff; visitor restriction; staff testing (50 negative); palivizumab to exposed infants; twice-weekly surveillance swabs	Attack rates 58.8% and 63.6%; 45.8% of infected infants asymptomatic; 20.8% required invasive ventilation (all <33 weeks); 3 deaths (not clearly RSV-attributed); shedding up to 29 days	Single NICU; descriptive; palivizumab effect not separable; abstract and full text differ (20 vs. 24 cases)
Rose et al. [6], USA	Outbreak investigation with WGS; level III NICU; December 2017	Not explicitly defined; relatedness by WGS (index case with onset 2 days after readmission classified as community-acquired)	7 RSV-positive infants in 11 days; 6/7 in one WGS cluster; unit census not reported	RT-PCR; WGS	Contact and droplet precautions; HCW symptom reporting; visitor screening, restriction and masking; hand hygiene; enhanced cleaning; staff cohorting; expanded surveillance	Transmission chains: ≥2 independent introductions; 3/7 infants required escalation of respiratory support; no deaths	Single outbreak; HCWs not tested; no attack rate
Vakrilova et al. [16], Bulgaria	Retrospective outbreak report; tertiary obstetric hospital (well-baby nursery and 45-bed NICU); February–March 2019	Not explicitly stated (nursery-born term infants symptomatic 2–5 days after discharge; NICU inpatients PCR-positive during stay)	21 infants (13 PCR-confirmed): 14/148 term infants from one nursery ward (6 confirmed) and 7/26 screened NICU infants (attack rate 26.9%)	Real-time RT-PCR (RSV-B)	Isolation and cohort nursing; PPE and hand hygiene; visitor restriction; exclusion of symptomatic staff/parents; screening of all NICU infants; palivizumab to all 26 exposed NICU infants	NICU: 6/7 LRTI, 2/7 mechanically ventilated, discharge delayed 1 week to >2 months, no deaths; 4/7 shed virus for 1–3 weeks	Retrospective; 8 term-infant cases not laboratory-confirmed; source of introduction not identified; no molecular linkage
Marcone et al. [19], Argentina	Retrospective analysis nested in a prospective study; two private hospitals, NICU-acquired vs. community-acquired ARI in infants <2 months; 2008–2011 and 2014–2016	ARI developing in infants never discharged since birth (no temporal threshold)	1 RSV among 20 NICU-acquired ARI (pathogen-positive 4/20) vs. 117 community-acquired ARI (RSV 52%)	Multiplex PCR (FilmArray)	Not reported	Pathogen positivity 20% in NICU-acquired vs. 95% in community-acquired ARI; no RSV-specific outcome	Single HA-RSV case; very small nosocomial group; sources not investigated
Aikphaibul et al. [20], Thailand	Retrospective cohort; tertiary pediatric wards; 2011–2016	RSV-LRTI diagnosed >72 h after admission	66/427 (15%) hospitalized RSV-LRTI in children <5 years	Rapid antigen test or PCR (plus ICD-10)	Not reported	HA vs. community-acquired: mortality 8% vs. 2%; ICU admission 24% vs. 12%; ventilation 27% vs. 10%; median LOS 43 vs. 6 days	Single center; ICD-10-based ascertainment; HA subgroup analyzed descriptively
Feldman et al. [21], USA	Retrospective surveillance with matched controls; PICU/ICUs, tertiary children’s hospital; 2005–2020	New viral RTI diagnosed ≥48 h after admission (ICU-acquired subset)	12 HA-RSV among 39 ICU-acquired high-risk viral RTIs (204 hospital-wide)	Viral culture, multiplex panel or PCR	Not reported	Outcomes pooled across viruses: LOS 56.9 vs. 4.8 days; escalation of respiratory support 61.5%; antibiotic escalation 33%; 2/39 deaths	Small single-center sample; outcomes not RSV-specific; pre-pandemic
Özen et al. [23], Turkey	Cross-sectional PCR study; PICU of a university hospital; September 2018–June 2021	Onset of respiratory symptoms ≥3 days after admission in children without respiratory signs at admission	8/40 (20%) children with suspected nosocomial RTI (vs. 7/60, 11.7%, community-acquired)	Multiplex real-time RT-PCR (nasopharyngeal swab)	Not reported	RSV is the most frequent virus in nosocomial RTI (20%); mean stay 22.6 vs. 10.7 days in nosocomial vs. community-acquired viral RTI (all viruses)	Small n; single PICU; tested children only, no incidence denominator; outcomes not RSV-specific
Pegoraro et al. [34], Italy	Before–after study (research letter); tertiary children’s university hospital; 20 September–23 November 2021 vs. 24 November 2021–28 February 2022	First positive RSV test >96 h after admission following a negative pre-admission or ≤96 h test (stored pre-admission swabs retested before screening began)	16 HA-RSV among 378 RSV-infected children: 11/151 (7.3%) before vs. 5/227 (2.2%) after universal pre-admission RSV RT-qPCR screening (*p* = 0.016; OR 3.49, 95% CI 1.19–10.25)	RT-qPCR	Combined SARS-CoV-2/RSV RT-qPCR before admission for all admitted children; repeat testing every 4th day in long-stay patients	Nosocomial RSV 7.3% → 2.2% (symptomatic children only: 7.3% → 1.6%); epidemic phase 11/6490 → 4/7740 patient-days (*p* = 0.038); no difference in sex, median age or ICU admission	Letter format; single center; consecutive short periods during an atypical epidemic, so secular trends not controlled
Saiman et al. [24], USA	Multicenter cohort (retrospective 2016–2019, prospective 2020–2021); six academic children’s hospitals	≥1 new or worsening respiratory symptom and positive RSV PCR ≥72 h after admission (or ≤48 h after a prior discharge)	122 HA-RSV cases over four seasons (median 22 per site); transmission index 0.66–1.38 HA-RSV per 100 hospitalized community-acquired RSV	PCR (multiplex panels)	Contact/droplet precautions initiated in 55.6% of cases on days 0–4; universal masking during 2020–2021 (transmission index did not decrease)	Escalation of respiratory support 45.1%; PICU transfer 14.8%; in-hospital death 4.1%; median post-diagnosis LOS 32 vs. 8 days with vs. without escalation	Academic children’s hospitals only; no community-acquired comparator; non-standardized testing
Most et al. [25], USA	Retrospective cohort; 490-bed pediatric hospital (all units); 2013–2018 (509,294 patient-days)	Positive test plus new respiratory symptom with onset after the virus-specific minimum incubation period (RSV: possible ≥4 days, definite ≥7 days); alternative definitions compared	RSV = 9.6% of 498 HA respiratory viral infections (≈48); RSV incidence 0.094 per 1000 patient-days (primary definition) vs. 0.251 with a >2-day definition	Multiplex PCR, Xpert Flu/RSV or antigen tests	Contact, droplet, and standard precautions; single rooms except semi-open NICU; mandatory staff influenza vaccination; monitored hand hygiene; visitor screening	Overall, HARVI incidence 0.98 per 1000 patient-days; incidence varied two-fold across definitions	Retrospective single center; RSV-specific outcomes not reported; pre-pandemic
Saiman et al. [31], USA	Prospective matched cohort; six academic children’s hospitals; October 2020–April 2022	New or worsening respiratory symptoms with positive RSV test ≥72 h after admission	26 HA-RSV children vs. 78 matched non-HA-RSV inpatients	Multiplex PCR	Not reported	Escalation of respiratory support: 39% vs. 18% (adjusted OR 5.1, 95% CI 1.4–19.1); 58% had ≥2 comorbidities	Academic centers only; residual confounding; small n
Sanders et al. [38], Australia	Retrospective before–after audit of the hospital HAI database; 359-bed tertiary children’s hospital; April 2019–March 2021	Infection does not present on admission and detected >48 h after admission, adjudicated by infection-control nurses	23 HA-RSV over 2 years (of 57 HA respiratory viral infections excluding rhinovirus); weekly medians 0	Multiplex PCR	Visitor restrictions from March 2020; universal surgical masks for staff and visitors from August 2020	All-virus HA-RVI fell from 10.8 to 0 per 10,000 bed-days (*p* = 0.004); HA-RSV decrease not significant (*p* = 0.34)	Very low event counts; concurrent interventions; clinician-directed testing
A2. Adult, geriatric, immunocompromised, and mixed-age settings							
Frange et al. [39], France	Prospective observational; 600-bed tertiary hospital (adult and pediatric units, 91% single rooms); October 2018–February 2019	RSV-ALRI with onset after 2–7 days (possible) or ≥8 days (definite) of continuous hospitalization	11 nosocomial RSV-ALRI (0.08% of inpatients; 0.17% of hospitalization days) vs. 229 community-acquired	PCR (nose/throat swabs, bronchial aspirate/BAL)	Droplet and contact precautions; annual staff training; hand and respiratory hygiene materials; visitor limitation in season; palivizumab for high-risk infants; cohorting	Nosocomial cases were more often adults (45.5% vs. 2.6%), and all had chronic complex conditions; longer LOS after onset (*p* < 0.0001); no deaths among nosocomial cases	One season; 11 cases; testing at clinician discretion; single tertiary center
Wong et al. [32], Hong Kong	Retrospective before–after surveillance; 3100-bed university hospital network; February–April 2017–2019 vs. 2020	Onset of fever or respiratory symptoms >48 h after admission	Nosocomial RSV: mean 3 ± 2 cases per month (0.04 per 1000 patient-days) before vs. 0 in February–April 2020 (*p* = 0.023)	Xpert Xpress Flu/RSV PCR	Universal masking of HCWs (100%) and patients (75.9%); pre-existing droplet precautions; hand hygiene compliance unchanged (73% vs. 77%)	Zero nosocomial RSV and influenza after universal masking	Three-month window; multiple concurrent COVID-19 measures; fewer patients tested >48 h after admission in 2020
Rangachar Srinivasa et al. [41], USA	Retrospective whole-genome-sequencing surveillance of healthcare-associated respiratory viruses; three teaching hospitals (one pediatric, two adult), Pittsburgh; January 2018–January 2020	Positive specimen from an inpatient hospitalized ≥3 days and/or with an inpatient or outpatient encounter within 30 days before the positive test	48 RSV-positive specimens (41 high-quality genomes); 3 genetically related RSV clusters (≤3 SNPs) of 2–4 patients; no denominator or attack rate	PCR; whole-genome sequencing	Not reported	RSV formed 3 of 14 clusters across four viruses; shared-unit links in 2 clusters, 2 clusters spanned pediatric and adult hospitals; RSV cluster time spans 8–55 days	Retrospective; case definition includes recent outpatient encounters; epidemiological links found for about half of clustered patients; no clinical outcomes; added from the update search
Hababou et al. [17], France	Outbreak investigation with genotyping; long-term geriatric hospital; March 2019	PCR-confirmed RSV ≥5 days after admission	19 RSV detections in 20 days; 12 on two adjacent wards formed the molecular cluster (7/7 sequenced identical); ward attack rate 35% (10/28)	Multiplex PCR; G-gene sequencing	Droplet and contact precautions; room confinement; staff PPE and hand hygiene; visitor restriction; suspension of communal activities; hygiene education	Clinical impact: lower-respiratory symptoms 7/12; cardiac decompensation 3/12; case fatality 2/12 (17%)	Retrospective; genotyping failed in ≈50% of samples; HCWs not investigated
Baier et al. [18], Germany	Outbreak investigation with target-capture WGS; adult hematology ward, university hospital; March 2019	RSV detected on day ≥5 of the ward stay after a negative admission test	4 RSV-B cases with identical genomes (28-bed ward; attack rate not reported)	RT-PCR with weekly prevalence screening; whole-genome sequencing	Admission and weekly PCR screening; immediate isolation; surgical masks for HCWs and visitors; hand hygiene retraining (compliance ≈ 70%)	All 4 asymptomatic detections; 1 LRTI requiring oxygen; no deaths; shedding up to 29 days	Very small outbreak; no denominator; index source unclear
Birrer et al. [33], Switzerland	Non-randomized pilot (bedside droplet precautions vs. single-room isolation); general internal medicine wards, university hospital; January–April 2019	New respiratory symptoms ≥day 3 after admission, PCR-confirmed	1 HA-RSV among 741 hospitalizations on DroPS wards (0 among 293 on regular wards); influenza/RSV HARVI 0.3% vs. 0.7%	Xpert Flu/RSV; multiplex PCR	Droplet precautions on-site in multi-bed rooms (bed signage, curtain, masks) vs. pathogen-based single-room isolation; daily symptom screening	Non-inferiority of DroPS for the combined influenza/RSV outcome (risk difference −0.4%, 95% CI −1.6 to 0.8)	Single HA-RSV case; non-randomized; one season; no post-discharge follow-up
Yamaguto et al. [22], Brazil	Retrospective cross-sectional analysis of active HAI surveillance records; tertiary teaching hospital; January 2013–December 2019	Pulmonary HAI per national (ANVISA 2017) criteria with a community respiratory virus detected; infections <72 h after admission excluded	35 RSV (21.7%) among 161 virus-positive pulmonary HAIs; 1174 pulmonary HAIs among 13,662 HAI reports (viruses tested in 544)	RT-PCR (nasal swab, tracheal aspirate, or BAL)	Not reported	Virus-positive pulmonary HAIs (all viruses): 76.4% in children; median stay 45 days (21 days to positivity); 7 deaths attributed to viral HAI, 3 of them with RSV	Viruses tested in about half of pulmonary HAIs; retrospective single center; outcomes not RSV-specific
Kim et al. [26], Republic of Korea	Prospective ICU cohort of severe pneumonia; 2700-bed tertiary hospital, medical ICU; 2010–2019	Pneumonia occurring ≥48 h after admission and not incubating on admission	44/92 (47.8%) severe hospital-acquired RSV pneumonias (vs. 39/163, 23.9%, for influenza); RSV caused 3.8% of 1276 severe HAP	Multiplex respiratory PCR	Not reported	90-day mortality 43.5% (all severe RSV pneumonia); hospital acquisition associated with mortality in univariate analysis of the pooled RSV/influenza cohort	ICU-only severe pneumonia; 33% of HAP cases not tested; no HA-specific outcome comparison for RSV
Hill-Ricciuti et al. [30], USA	Retrospective matched cohort within prospective surveillance; two academic medical centers; 2017–2020 (three seasons)	RSV detected by PCR ≥4 days after admission (community-onset ≤3 days)	84 HA-RSV adults vs. 160 matched community-onset patients; no clusters identified	PCR	Not reported	LOS after detection 10 vs. 6 days; higher level of care at discharge 44% vs. 14% (adjusted OR 7.0); escalation of respiratory support in 29%; in-hospital death 15% vs. 6% (NS)	Two centers; non-systematic testing; no cost data
Ehrenzeller et al. [35], USA	Interrupted time-series analysis; 803-bed academic adult hospital; October 2015–April 2023	Virus first detected on hospital day ≥4 (sensitivity analysis ≥5)	84 hospital-onset RSV among 436 hospital-onset respiratory viral infections over 8 years (19.3%)	PCR or antigen tests	From March 2020: universal masking, staff symptom attestation, visitor restrictions, patient symptom screening	Hospital-onset influenza/RSV 53% lower post-pandemic than pre-pandemic after adjustment for community activity (RR 0.47)	Single hospital; RSV pooled with influenza in effect estimates; testing practices changed over time
Wilson Dib et al. [36], USA	Retrospective before–after surveillance; 767-bed comprehensive cancer center (single rooms); September 2016–February 2020 vs. March 2020–March 2022	Respiratory PCR positive ≥5 days after admission for RSV (institutional virus-specific criterion) or within a defined window after discharge and readmission	Rates only: nosocomial RSV 0.51 → 0.15 per 1000 admissions (IRR 0.30, 95% CI 0.15–0.61); absolute RSV case numbers not reported	Respiratory PCR	Facility-wide medical masks for staff, patients and visitors; eye protection during surges; admission SARS-CoV-2 PCR; no-visitor policy March 2020–June 2021; entry screening and hand hygiene	All nosocomial respiratory viral infections 5.24 → 1.90 per 1000 admissions (IRR 0.36); nosocomial RSV incidence higher during COVID-19 community surges	Ecological before–after design; admissions as denominator; no RSV case counts or RSV-specific outcomes
Chen et al. [27], Taiwan	Retrospective case series of severe pneumonia tested with a pneumonia PCR panel; medical center; August 2021–June 2023	Positive RSV test with ≥48 h between admission and symptom onset	4/14 severe RSV pneumonias hospital-acquired (a further 4 healthcare-associated); denominator not reported	Multiplex pneumonia PCR panel	Not reported; no outbreak investigation	Hospital mortality 7/14 (50%); 1/4 nosocomial cases died; bacterial co-infection 71%	Very small series; selection bias (panel used only for severe pneumonia); no denominator
Birrer et al. [37], Switzerland	Prospective single-arm surveillance; medical wards of a tertiary and a secondary hospital; January–March 2020	New respiratory symptoms ≥3 days after admission to a DroPS ward, PCR-confirmed	2 HA-RSV (plus 1 influenza B) among 764 at-risk patients in multi-bed rooms (0.4% confirmed HARVI)	PCR (Cobas Liat influenza/RSV)	Symptom-triggered droplet precautions on-site in multi-bed rooms; no contact isolation for RSV; immunosuppressed patients not co-roomed	No transmission source identified for either RSV case; no HARVI among 241 patients exposed to an infected room neighbour	Single arm; truncated by COVID-19; no asymptomatic screening
Auvinen et al. [28], Finland	Retrospective cohort; adult wards of two acute-care hospitals; 2016–2022	ILI onset ≥5 days after admission (confirmed); tested after minimum incubation (possible)	12/432 (2.8%) RSV-positive adults (vs. 122/1500, 8.1%, for influenza); 0.027 vs. 0.32 per 1000 bed-days	RT-PCR (rapid antigen test for influenza until 2019)	Standard precautions only for RSV (droplet precautions for influenza); universal staff masking from March 2020	Incidence and LOS: 0.027 (95% CI 0.013–0.050) per 1000 bed-days; median LOS 20 vs. 12 days (nosocomial influenza); virus-specific ICD-10 code in 17%	Retrospective; onset after discharge missed; transplant recipients excluded; no mortality data
Gettler et al. [40], USA	Retrospective analysis of population-based surveillance (RSV-NET); acute-care hospitals in an 8-county area; 2016–2019 seasons	Traditional: positive test after hospital day 3; expanded: plus, positive test within 3 days in patients transferred from post-acute care or discharged ≤7 days earlier	41/900 (4.6%) laboratory-confirmed RSV hospitalizations (traditional) and 136/900 (15.1%) (expanded); incidence 0.8 vs. 2.65 per 100,000	Molecular assay or rapid antigen test	Not reported	Traditional HA-RSV: median LOS 9 vs. 4 days for all RSV hospitalizations; ICU admission 32% vs. 21%	Classification by test date without symptom review; single region; no mortality data
Salachas et al. [29], France	Retrospective ward-level cohort; 900-bed tertiary hospital, 27 adult wards; 2014–2023	Positive PCR on a sample collected ≥5 days after admission	95 HA-RSV among 2318 HA viral respiratory infections (4.05%) and 513 RSV detections; overall HA-VRI 0.57 per 1000 hospital-days	Multiplex, duplex or monoplex PCR	Ward-level hand-sanitizer and mask consumption; percentage of double-occupancy rooms (40%); admission SARS-CoV-2 screening from 2020	HA-VRI incidence rose with ward community-acquired VRI incidence and with the proportion of double-occupancy rooms (+0.04 per 1000 hospital-days per 10%)	Ecological ward-level analysis; test-date definition; SARS-CoV-2 dominates HA-VRI; RSV-specific outcomes not analyzed
Part B. Hospital-based study without a reported healthcare-associated subset (contextual evidence)							
Chaiut et al. [42], Thailand	Retrospective observational; tertiary pediatric wards; 2016–2021	Not defined; nosocomial subset not reported. Retained as contextual evidence on post-pandemic seasonality and clinical presentation	None; 358 hospitalized RSV-LRTI (284 in 2016–2019; 74 in 2020–2021)	RT-PCR or rapid antigen test	Not reported (community NPIs described)	Seasonality and presentation before vs. during the pandemic: 2020 season started later (October) and was shorter; fever on admission 69% → 50%; median age 1 → 2 years	Single center; RSV not tested in all respiratory admissions; ICD-10-based inclusion

RSV, respiratory syncytial virus; HA-RSV, healthcare-associated RSV; NICU, neonatal intensive care unit; PICU, pediatric intensive care unit; ICU, intensive care unit; ARI, acute respiratory infection; LRTI/ALRI, (acute) lower respiratory tract infection; RTI, respiratory tract infection; ILI, influenza-like illness; HAI, healthcare-associated infection; HARVI/HA-VRI, healthcare-associated (viral) respiratory infection; HAP, hospital-acquired pneumonia; DroPS, droplet precautions on-site; PCR, polymerase chain reaction; RT-PCR, reverse-transcription PCR; WGS, whole-genome sequencing; HCW, healthcare worker; IPC, infection prevention and control; LOS, length of stay; NPI, non-pharmaceutical intervention; OR, odds ratio; CI, confidence interval. Study period denotes the period of data collection. “Not reported” indicates that the item was not described in the source. Percentages and medians are quoted as reported by the source; comparator groups are RSV-negative matched controls (Comas-García), community-acquired or community-onset RSV (Aikphaibul, Frange, Hill-Ricciuti, Auvinen [influenza]), matched non-HA-RSV inpatients (Saiman 2024), or the pre-intervention period (before–after studies).

#### 3.1.1. Neonatal and Pediatric Settings

Neonatal units contributed three outbreak investigations and two cohort studies. In a Brazilian NICU, two consecutive outbreaks caused by different RSV-A genotypes infected 10 of 17 and 14 of 22 infants (attack rates 58.8% and 63.6%); 46% of infected infants were asymptomatic, 21% required invasive ventilation (all born before 33 weeks), and shedding persisted for up to 29 days [15]. In a Bulgarian obstetric hospital, an outbreak that began on a well-baby nursery ward, where 14 of 148 term infants became symptomatic within days of discharge, spread to the NICU, where 7 of 26 screened preterm infants were infected (26.9%), 2 required mechanical ventilation, and discharge was delayed by one week to more than two months [16]. In a U.S. level III NICU, whole-genome sequencing of seven RSV-positive infants identified within 11 days showed that six formed a single cluster, whereas the index case was a separate community introduction; three infants required escalation of respiratory support, and none died, and because healthcare workers were not tested, the source of introduction could not be established, although ill-visitor exposure was documented [6]. In the Mexican NICU cohort, 24 infants with RT-PCR-confirmed HA-RSV (2012–2017) remained in hospital for a median of 24 days after RSV detection compared with 13 days in matched RSV-negative controls (total stay 44 vs. 35.5 days) and, after detection, more often received antibiotics (45.8% vs. 8.3%), required mechanical ventilation, and developed a further nosocomial infection (45.8% vs. 0%); median direct costs after RSV detection were US $3587 per infant compared with US $1124 in controls, and total hospital charges were 41.9% (≈US $1836 per infant) higher [4]. By contrast, in two Argentinian private hospitals, only 1 of 20 NICU-acquired respiratory infections in infants younger than two months was due to RSV, illustrating the variability of HA-RSV between units [19].

In pediatric wards and PICUs, the largest dataset is the multicenter surveillance of six U.S. academic children’s hospitals, which identified 122 sporadic HA-RSV cases over four seasons (positive test ≥72 h after admission), corresponding to a transmission index of 0.7–1.4 HA-RSV cases per 100 hospitalized community-acquired cases; 45% required escalation of respiratory support, 15% were transferred to the PICU, and 4% died in hospital [24]. In the subsequent prospective matched cohort from the same hospitals (October 2020–April 2022), 26 children with HA-RSV were compared with 78 matched inpatients without HA-RSV; 39% versus 18% required escalation of respiratory support (adjusted odds ratio 5.1, 95% CI 1.4–19.1) [31]. In the Thai tertiary-center cohort of 427 children younger than 5 years hospitalized with RSV lower respiratory tract infection, 66 (15%) acquired the infection more than 72 h after admission; compared with community-acquired cases, HA-RSV was associated with higher mortality (8% vs. 2%), more frequent ICU admission (24% vs. 12%) and ventilatory support (27% vs. 10%), and a markedly longer median stay (43 vs. 6 days), although the long stay partly reflects the underlying conditions that led to prolonged hospitalization [20]. In a single-center PICU surveillance study spanning 2005–2020, RSV accounted for 12 of 39 ICU-acquired high-risk viral respiratory infections (≥48 h after admission), with outcomes reported for all viruses combined (length of stay 56.9 vs. 4.8 days in controls; escalation of respiratory support in 61.5%) [21], and in a Turkish PICU RSV was the most frequently detected virus among children with suspected nosocomial respiratory infection (symptom onset ≥3 days after admission; 8/40, 20%) [23]. Two studies quantified incidence and its dependence on the case definition: in a 490-bed U.S. children’s hospital the incidence of HA-RSV was 0.094 per 1000 patient-days when onset was required to occur after the virus-specific minimum incubation period (≥4 days) but 0.251 per 1000 patient-days when symptom onset after hospital day 2 was counted regardless of the virus-specific incubation period [25], and in an Australian tertiary children’s hospital 23 HA-RSV cases were recorded over two years, with weekly counts too low to detect an effect of pandemic-era visitor restrictions and universal masking on RSV specifically, although all-virus healthcare-associated respiratory infections fell to zero [38]. Finally, in an Italian children’s hospital, combined SARS-CoV-2/RSV RT-qPCR screening before admission, introduced in November 2021 during an early and unexpected RSV epidemic, was followed by a fall in nosocomial RSV (first positive test >96 h after admission following a negative earlier test) from 11 of 151 (7.3%) to 5 of 227 (2.2%) RSV-infected children (*p* = 0.016; odds ratio 3.49) [34].

#### 3.1.2. Adult, Geriatric, Immunocompromised, and Mixed-Age Settings

In adult acute-care hospitals, HA-RSV is less frequent than in children but consistently under-recognized. In two Finnish hospitals, 12 of 432 RSV-positive adult inpatients (2.8%) met the definition of nosocomial infection (influenza-like illness onset ≥5 days after admission), corresponding to an incidence of 0.027 (95% CI 0.013–0.050) cases per 1000 bed-days, roughly one-tenth of the incidence of nosocomial influenza in the same hospitals, where RSV-positive patients were managed with standard precautions only and most rooms were multi-occupancy; nosocomial cases were older (75% aged ≥65 years), clustered on internal-medicine wards, had a median stay of 20 days, and carried a virus-specific diagnosis code in only 17% of cases [28]. In a matched cohort from two U.S. academic centers (2017–2020), 84 adults with HA-RSV (PCR-positive ≥4 days after admission) had a longer stay after detection than 160 community-onset patients (median 10 vs. 6 days), were more likely to require a higher level of care at discharge (44% vs. 14%; adjusted odds ratio 7.0), and 29% required escalation of respiratory support, whereas ICU admission and mechanical ventilation did not differ [30]. Population-based surveillance in Tennessee found that 4.6% of 900 laboratory-confirmed RSV hospitalizations met a traditional hospital-onset definition (positive test after hospital day 3) and 15.1% met an expanded definition that also captured patients transferred from post-acute care or recently discharged, with a longer median stay (9 vs. 4 days) and a higher ICU admission rate (32% vs. 21%) among hospital-onset cases [40]. In a 10-year ward-level surveillance study in a 900-bed Paris hospital, RSV accounted for 95 of 2318 healthcare-associated viral respiratory infections (4%), and the incidence rose with community activity and the proportion of double-occupancy rooms on the ward [29]. Among critically ill adults with severe pneumonia in a Korean medical ICU, 44 of 92 severe RSV pneumonias (47.8%) were hospital-acquired, twice the proportion observed for influenza, and RSV accounted for 3.8% of severe hospital-acquired pneumonia over nine seasons; 90-day mortality was 43.5% (40/92) in the severe RSV pneumonia cohort as a whole and was not reported separately for the hospital-acquired subgroup [26]; in a Taiwanese medical center, 4 of 14 severe RSV pneumonias identified with a pneumonia PCR panel were hospital-acquired [27].

Four studies from mixed adult–pediatric settings add complementary information. In three teaching hospitals in Pittsburgh (one pediatric, two adult), retrospective whole-genome sequencing of respiratory viruses from patients hospitalized for at least three days or with a recent healthcare encounter yielded 41 high-quality RSV genomes and identified three genetically related RSV clusters of two to four patients; shared unit stays linked patients in two clusters, and two clusters included patients from both the pediatric and an adult hospital [41]. In a Paris tertiary hospital with 91% single rooms, 11 nosocomial RSV lower respiratory tract infections occurred in one season (0.08% of inpatients); all affected patients had chronic complex conditions, and adults were over-represented among nosocomial compared with community-acquired cases (45.5% vs. 2.6%) [39]. In a Hong Kong hospital network, nosocomial RSV averaged three cases per month (0.04 per 1000 patient-days) in February–April of 2017–2019 and fell to zero in February–April 2020 after universal masking of staff and patients was introduced [32]. In a Brazilian teaching hospital, RSV accounted for 35 (21.7%) of 161 virus-positive pulmonary healthcare-associated infections (onset ≥72 h after admission) recorded over seven years, three-quarters of them in children [22].

Immunocompromised and geriatric patients were the subject of three studies. On an adult hematology ward in Germany, weekly PCR screening detected a cluster of four RSV-B infections that target-capture sequencing confirmed to be monoclonal; all four patients were asymptomatic at detection, one developed lower respiratory tract infection, and shedding persisted for up to 29 days [18]. At a U.S. comprehensive cancer center, the incidence of nosocomial RSV (positive test ≥5 days after admission) fell from 0.51 to 0.15 per 1000 admissions (incidence rate ratio 0.30; 95% CI 0.15–0.61) after pandemic-era infection-prevention measures were introduced and was higher during community surges [36]. In the French long-term geriatric hospital outbreak, 19 RSV detections occurred within 20 days among patients hospitalized for a mean of 56 days; 12 cases on two adjacent wards constituted the molecular cluster (ward attack rate 35%), 7 of 12 had lower-respiratory symptoms, 3 developed cardiac decompensation, and 2 (17%) died [17]. Two Swiss studies evaluated bedside droplet precautions in multi-bed rooms as an alternative to single-room isolation on general medical wards and recorded one and two HA-RSV cases, respectively, among 741 and 764 at-risk hospitalizations, without identifying a transmission source in the latter [33,37]. Finally, in an 803-bed U.S. academic hospital, 84 hospital-onset RSV infections were detected over eight years, and hospital-onset influenza and RSV combined were 53% lower after the pandemic than before it, after adjustment for community activity, in the presence of universal masking, staff symptom attestation, and visitor restrictions [35].

#### 3.1.3. Synthesis: Incidence, Attributable Burden, and Certainty of Evidence

Across the core studies, HA-RSV accounted for 2.8% of RSV-positive adult admissions [28], 4.6–15.1% of laboratory-confirmed RSV hospitalizations depending on the definition [40], and 15% of pediatric RSV lower respiratory tract infections in a tertiary center [20]; reported incidences ranged from 0.027 per 1000 bed-days in adults [28] to 0.094–0.251 per 1000 patient-days in children, depending on the case definition [25]. The temporal criterion strongly affected measured incidence—a 2.7-fold difference between incubation-based and 48 h definitions in one children’s hospital [25] and a 3.3-fold difference between traditional and expanded definitions in population-based surveillance [40]—which underscores the need for standardized surveillance definitions. The core studies consistently show that HA-RSV is associated with prolonged hospitalization (a median of 11 additional days in hospital after RSV detection versus matched controls in the NICU cohort [4]; 10 vs. 6 days after detection in adults [30]; median stays of 43 days in Thai children and 20 days in Finnish adults with nosocomial infection [20,28]), escalation of respiratory support in approximately 30–45% of affected patients [24,30,31], higher antibiotic use and secondary infections in neonates [4], a need for a higher level of care at discharge in adults [30], and, in children, higher mortality than community-acquired infection [20]. Excess costs associated with HA-RSV were quantified in only one core study (total hospital charges 41.9%, or ≈US $1836 per infant, higher than in matched NICU controls [4]); an earlier matched case–control study from a U.S. children’s hospital estimated an attributable cost of US $9419 per nosocomial case and a return of approximately US $6 for every US $1 spent on infection control [43]. At the level of systematic-review evidence, a 2025 meta-analysis of 27 studies estimated a pooled HA-RSV incidence of 10.86 cases per 1000 person-years among hospitalized children, a pooled case-fatality rate of 13.3%, and that HA-RSV accounted for 15.6% of pediatric RSV hospitalizations, with significantly higher mortality than community-acquired infection [44]; a global case series of RSV-related in-hospital deaths found that children dying of nosocomial RSV were more often preterm (46% vs. 24%) or had comorbidities (87% vs. 57%) than those dying of community-acquired infection [45].

Eight core studies provide information on the effect of infection-prevention measures on HA-RSV: the seven evaluations described above [32,33,34,35,36,37,38] and the multicenter pediatric cohort [24]. Universal masking with other pandemic-era measures was followed by the disappearance of nosocomial RSV in Hong Kong [32], a 70% reduction at a U.S. cancer center [36], and a 53% reduction in hospital-onset influenza and RSV combined in a U.S. academic hospital [35], whereas in six U.S. children’s hospitals the HA-RSV transmission index did not fall during universal masking in 2020–2021 [24] and in an Australian children’s hospital the RSV-specific effect could not be demonstrated because of low counts [38]. Universal pre-admission PCR screening was followed by an approximately 70% reduction in the proportion of nosocomial RSV infections (7.3% to 2.2%) in one Italian children’s hospital [34], and symptom-triggered bedside droplet precautions in multi-bed rooms were not inferior to single-room isolation for the combined influenza/RSV outcome in one Swiss pilot study, albeit with very few RSV events [33,37]. These observational designs cannot separate the effects of individual measures from concurrent changes in community RSV circulation and testing.

The certainty of this evidence is limited. Twenty of the 29 core studies were single-center; most were retrospective; sample sizes of HA-RSV cases ranged from 1 to 136; case definitions and denominators differed; and only three studies compared RSV-specific clinical or economic outcomes against a matched comparison group [4,30,31]. Several further core studies used matching, adjustment, or formal effect estimates, but pooled RSV with other respiratory viruses [21,35] or used infection rates rather than patient outcomes as the endpoint [33,36]. Outbreak investigations describe transmission under specific circumstances and cannot yield incidence estimates, and before–after studies of pandemic-era measures are confounded by the concurrent collapse of community RSV circulation. The Discussion addresses these limitations, interpreting the core findings in light of contextual evidence.

### 3.2. Contextual Evidence: Evolving Epidemiology in the Post-Pandemic Era

The evidence in this section derives from population-based surveillance, systematic reviews, and modeling studies of RSV in general populations; it describes the epidemiological background in which HA-RSV occurs and is not evidence on HA-RSV itself.

#### 3.2.1. Temporal Dynamics: From Seasonality to Observed and Potential Post-Pandemic Overlap

Before the pandemic, HA-RSV tracked the predictable seasonal pattern of community circulation, informing staffing, palivizumab scheduling, and surge-bed planning. In neonatal and pediatric units, early outbreak investigations documented secondary attack rates of 20–45% among exposed infants when protocols lapsed, and infection of a substantial proportion of ward staff (56% in one early report), some of whom continued to work while mildly symptomatic [2,3]. In adult acute-care hospitals, the reported incidence of nosocomial RSV was 0.027 cases per 1000 bed-days, although underdiagnosis and incomplete coding were common [28].

This equilibrium collapsed in 2020. Widespread non-pharmaceutical interventions (NPIs) against SARS-CoV-2—masking, school closures, visitor bans, and enhanced hand hygiene—were accompanied by unprecedentedly low RSV detection across Europe, North America, and parts of Asia, effectively eliminating the 2020–21 winter season (systematic-review evidence [9,46]). Mathematical models forecast that such suppression would create an “immunity debt”: a temporary accumulation of susceptible children and adults whose routine viral exposures had been postponed [10].

Off-season resurgences did occur once NPIs relaxed. From mid-2021, many countries saw sudden off-season epidemics—some in early summer or late spring—with peaks matching or exceeding the prior decade. In an Ontario population-based cohort, the 2021–2022 RSV season peaked slightly earlier than expected, while the 2022–2023 season peaked about one month earlier and produced more than twice as many pediatric hospitalizations as prepandemic seasons [47]. In the United States, the 2021–2022 epidemic began 21 weeks earlier than prepandemic seasons and peaked in July [48]. Immunity debt is one explanation for these observations, and the loss of regular community boosting has been proposed to have eroded adult antibody levels and raised the risk of severe disease in older people [10]; measured declines in RSV-specific antibody in women of child-bearing age and infants during the RSV-free year support this mechanism [13]. However, the accumulation of RSV-naïve infants across two birth cohorts, changes in testing intensity and health-seeking behavior, viral interference between SARS-CoV-2 and RSV, the emergence of a genetically distinct RSV-A lineage in some regions, and climatic factors have also been proposed, and the observed changes most likely reflect several interacting factors whose relative contributions have not been quantified [7,8,11,12,14]. During such surges, HCWs and visitors with asymptomatic or mild infection can act as under-recognized sources of onward transmission [3].

**Figure 2:**
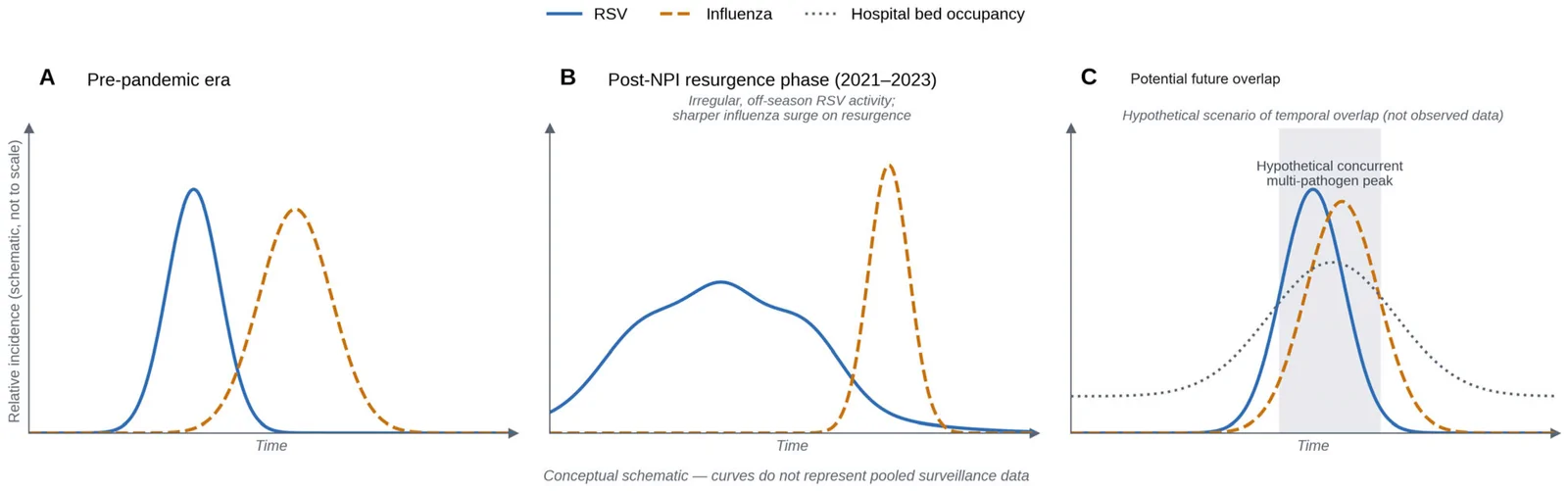
Conceptual schematic of RSV and influenza temporal dynamics: from sequential seasonality to potential post-pandemic overlap. This figure is a conceptual illustration; the curves are schematic, depict relative incidence (not to scale), and do not represent pooled surveillance data or a formal comparative analysis. Panels (**A**–**C**) correspond, from left to right, to the three phases described in the text. (**A**) Pre-pandemic era: sequential winter waves, with RSV peaks preceding influenza epidemics by a distinct interval (1–2 months). (**B**) Post-NPI resurgence phase (2021–2023): RSV exhibited temporal shifts with irregular, off-season epidemics, whereas influenza exhibited an intensity shift with sharper, higher-magnitude surges upon resurgence. (**C**) Potential future overlap: recent seasons (2023–2025) have shown a gradual return toward winter seasonality; the panel depicts a hypothetical scenario of temporal overlap between RSV and influenza waves that could produce a concentrated multi-pathogen surge (shaded area) coinciding with peak hospital bed occupancy. Panels (**A**,**B**) are informed by U.S. RSV surveillance and recent influenza ecological analyses [48,50]; panel (**C**) is a projected scenario, not an observed trend.

As the acute phase of the pandemic recedes, surveillance from 28 countries indicates that recent seasons have largely returned toward winter seasonality, although substantial interannual variation remains [48,49]. Concurrent circulation of RSV, influenza, and SARS-CoV-2 was observed in late 2022, and shorter intervals or overlap between RSV and influenza activity may concentrate hospital demand during respiratory-virus seasons. Whether such overlap will recur is not established; we present it here as a scenario hospitals should plan for rather than as an observed trend and depict it conceptually in Figure 2.

#### 3.2.2. Geographical Heterogeneity and Climatic Drivers

**Figure 3:**
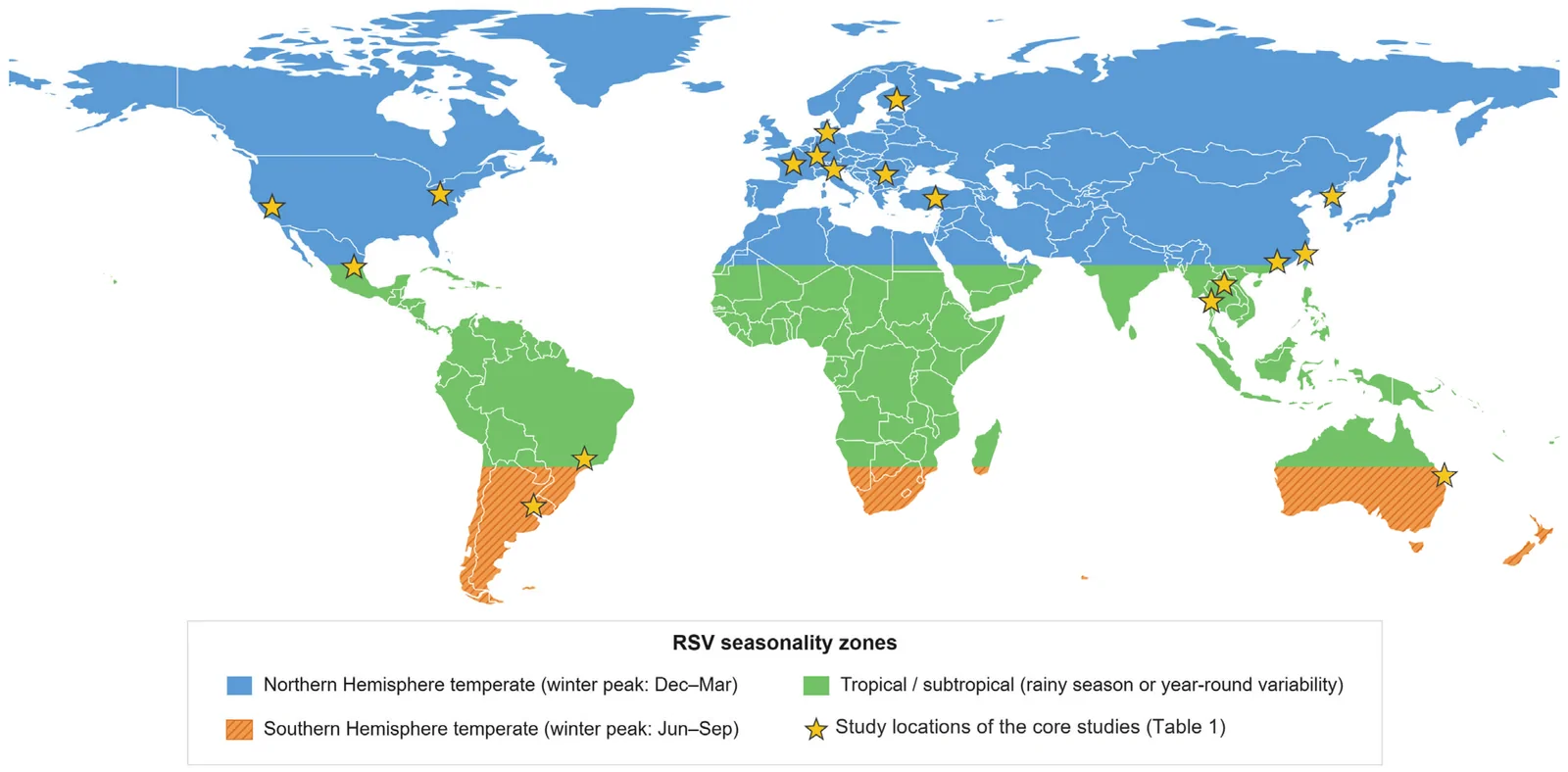
Conceptual schematic of the global distribution of RSV seasonality and geographic distribution of the primary studies included in Table 1; the figure does not represent pooled quantitative estimates or a formal comparative analysis. The map shows a simplified latitude-based classification of three primary RSV seasonality patterns: temperate Northern Hemisphere regions (classic winter peaks, December–March); temperate Southern Hemisphere regions (winter peaks, June–September); and tropical/subtropical regions (association with the rainy season or year-round variability). Gold stars indicate the countries and territories in which the primary studies of Table 1 were conducted; markers are placed at approximate coordinates rather than at the exact institutions, and several countries contributed more than one study. Seasonality classifications provide a simplified conceptual representation of broad RSV seasonality patterns, informed by global reviews and U.S. surveillance data [46,48]; the base map is derived from public-domain Natural Earth data. While post-pandemic surges (2021–2023) frequently deviated from these patterns, recent data suggest a return toward these baseline classifications.

The pandemic exposed the virus’s susceptibility to behavioral and climatic influences, producing marked geographical heterogeneity. Figure 3 delineates these seasonality zones and the geographic distribution of the primary studies included in Table 1.

Southern Hemisphere regions (e.g., Australia, South Africa) acted as early indicators of disruption. After a near-complete disappearance in 2020, they underwent a pronounced out-of-season resurgence in late 2020 and early 2021 (austral summer/autumn), demonstrating that, absent population immunity, RSV can sustain epidemic transmission even under atypical climatic conditions [49].

In the Northern Hemisphere (e.g., USA, Europe, Japan), the pattern was inverted. Following a “silent winter” in 2020–2021, an unprecedented summer surge occurred; U.S. CDC data showed a historic peak in July 2021—a marked deviation from the conventional winter pattern [48].

Tropical and subtropical regions (e.g., Thailand, Taiwan), traditionally governed by rainy-season patterns, were similarly disrupted. In Thailand, a single-center study of 358 hospitalized children found that the 2020 season began later and was shorter than pre-pandemic seasons and that clinical presentation changed: fever on admission fell from 69% to 50%, and the median age rose from 1 to 2 years, although the study did not distinguish nosocomial from community-acquired cases [42].

Recent seasons suggest a gradual return toward winter activity, although the timing of epidemic onset and peak activity remains variable across regions. Hospitals should therefore align infant immunoprophylaxis with local RSV surveillance and national guidance.

### 3.3. Modes of Transmission

Respiratory particles expelled during coughing, sneezing, talking, and breathing form a continuum of sizes rather than two discrete classes, and the traditional 5 µm boundary between “droplets” and “aerosols” is a historical convention that does not define distinct transmission routes [51,52]. What is established for RSV, from volunteer studies, outbreak investigations, and early experimental work, is that transmission occurs predominantly at short range—through larger respiratory particles that settle within 1–2 m and through direct and indirect contact with secretions—rather than over long distances [3,53]. In the classic volunteer study, infection occurred among those who had close contact with infected infants (“cuddlers”) and those who touched contaminated surfaces (“touchers”), but not among those who sat more than 1.8 m away without contact [53]. This short-range predominance accounts for the high secondary attack rates among exposed roommates when precautions lapse [3].

Contact transmission via contaminated hands and fomites is therefore a major pathway (experimental and outbreak evidence): infectious RSV was recovered from countertops for up to 6 h, from rubber gloves for up to 1.5 h, from gowns and tissues for 30–45 min, and from skin for up to 20 min in early experiments [54], and viable virus has been recovered from stainless steel after several days in a recent in vitro study [55]. Hand hygiene and prompt disinfection of high-touch objects (bed rails, monitors, and stethoscopes) are therefore as important as masking, and direct contact during routine care adds further risk.

Transmission through smaller airborne particles is biologically plausible but not established as a route of transmission for RSV: infectious RSV has been detected in particles <5 µm in the air surrounding infants with bronchiolitis [56] (mechanistic evidence), and RSV RNA was detected in 35 of 200 air samples from a pediatric emergency-department corridor over one year, with concentrations tracking the RSV season [57] (environmental detection, not evidence of infectivity or transmission), but epidemiological evidence of long-range airborne spread is lacking. Recommendations concerning aerosol-generating procedures (open suctioning, non-invasive ventilation, and bronchoscopy) are therefore guidance-based rather than evidence of an airborne route: CDC isolation guidance assigns RSV to Standard and Contact Precautions and does not require airborne infection isolation rooms for its routine care [58]; during aerosol-generating procedures, the level of respiratory protection and the choice of room are determined by the pathogens suspected, the procedure, the possibility of co-infection with an airborne-transmitted pathogen, and institutional policy, rather than by an RSV-specific requirement [58]; the respirator requirement in SARS-CoV-2 guidance [59] applies to that pathogen and does not transfer to RSV.

Symptomatic patients are the primary source, shedding high viral loads for six to seven days—and beyond three weeks in immunocompromised hosts [3]. Asymptomatic or mildly symptomatic infection is a further, less visible source: in an early outbreak investigation, 56% of exposed ward staff became infected, and roughly one in five infected staff had no symptoms [2]; prospective PCR surveillance of on-duty HCWs has detected respiratory viruses in asymptomatic staff [60], including RSV in a UK healthcare-worker cohort in which 37.5% of RSV infections were asymptomatic [61]; and whole-genome sequencing has traced multiple independent introductions into NICUs, shared strains among patients and staff on an adult stem-cell transplant unit, and small genetically linked RSV clusters across hospital units [6,41,62]. Researchers also identified asymptomatic nursing staff during a genotyped neonatal outbreak [63]. These data show that HCWs can introduce and transmit RSV; however, they do not quantify their contribution relative to patients, visitors, caregivers, or repeated community introductions [64]. Shared equipment and high-touch surfaces act as reservoirs that perpetuate self-inoculation where cleaning and hand hygiene lapse [3].

### 3.4. Vulnerable Hosts: The Gradient of Clinical Severity

Inpatient groups experience disproportionate morbidity from HA-RSV. The evidence below combines the core HA-RSV studies with contextual data from general hospitalized RSV populations; the latter are labeled as such and describe the vulnerability of these groups to RSV disease rather than the attributable burden of healthcare-associated infection.

#### 3.4.1. Neonates

Neonates and young infants are most vulnerable; prematurity, incomplete maternal antibody transfer, and narrow airways amplify the risk. In the Mexican NICU cohort (core evidence), infants with HA-RSV remained in hospital for a median of 11 days longer after RSV detection than matched controls and, after detection, received antibiotics 5.5 times more often [4]. Secondary attack rates approaching 45% have been reported when index cases were not isolated promptly (early outbreak reports summarized in [3]). In a global case series of RSV-related in-hospital deaths, children dying of nosocomial infection were more often preterm or had comorbidities than those dying of community-acquired infection [45].

#### 3.4.2. Children

Children with complex medical needs—congenital heart disease, chronic lung disease, or neuromuscular disorders—experience marked escalation in severity once infected. In the multicenter U.S. cohort (core evidence), 58% of children with HA-RSV had two or more comorbidities and 39% required escalation of respiratory support [31]; in the Thai cohort, HA-RSV carried a fourfold higher mortality than community-acquired infection [20]. As contextual evidence from general PICU populations (not restricted to HA-RSV), RSV-positive encounters accounted for 11.4% of U.S. pediatric ICU encounters and 21.1% of ICU days in 2017–2023 and were more often associated with positive-pressure ventilation than RSV-negative encounters (29.7% vs. 17.7%) [65].

#### 3.4.3. Immunocompromised Patients

Immunocompromised patients, particularly HSCT and solid-organ transplant recipients, may experience prolonged viral shedding and progression to lower-respiratory disease [3]. Whole-genome sequencing has documented nosocomial spread of a single RSV strain among patients and staff on an adult stem-cell transplant unit [62]. As contextual evidence from a general (not nosocomial-specific) hematology population, at an Italian tertiary hematology and HSCT center, 24 of 54 patients with RSV infection (44%) progressed to lower-respiratory-tract infection; the overall case-fatality rate was 11%, and RSV-related mortality was 5.5% [66].

#### 3.4.4. Older Adults and Frailty

Older adults now rival pediatric wards as consumers of hospital resources. As contextual evidence from population-based surveillance of all RSV admissions, surveillance in 12 U.S. states (RSV-NET, 2022–2023) found that patients aged ≥75 years accounted for over half of RSV admissions, with 17% originating in long-term-care facilities [67]. In-hospital case-fatality rates among older adults vary substantially by age and geographic setting and may reach approximately 9% in some resource-limited settings [68]. Co-existing chronic obstructive pulmonary disease (COPD) or heart failure further raises the odds of ICU admission [67]. Direct evidence on HA-RSV in this group comes from the Finnish cohort, in which three-quarters of nosocomial cases were aged ≥65 years [28], the U.S. adult matched cohort, in which HA-RSV was associated with a need for a higher level of care at discharge [30], and the French geriatric outbreak, in which 2 of 12 cluster cases died [17].

**Figure 4:**
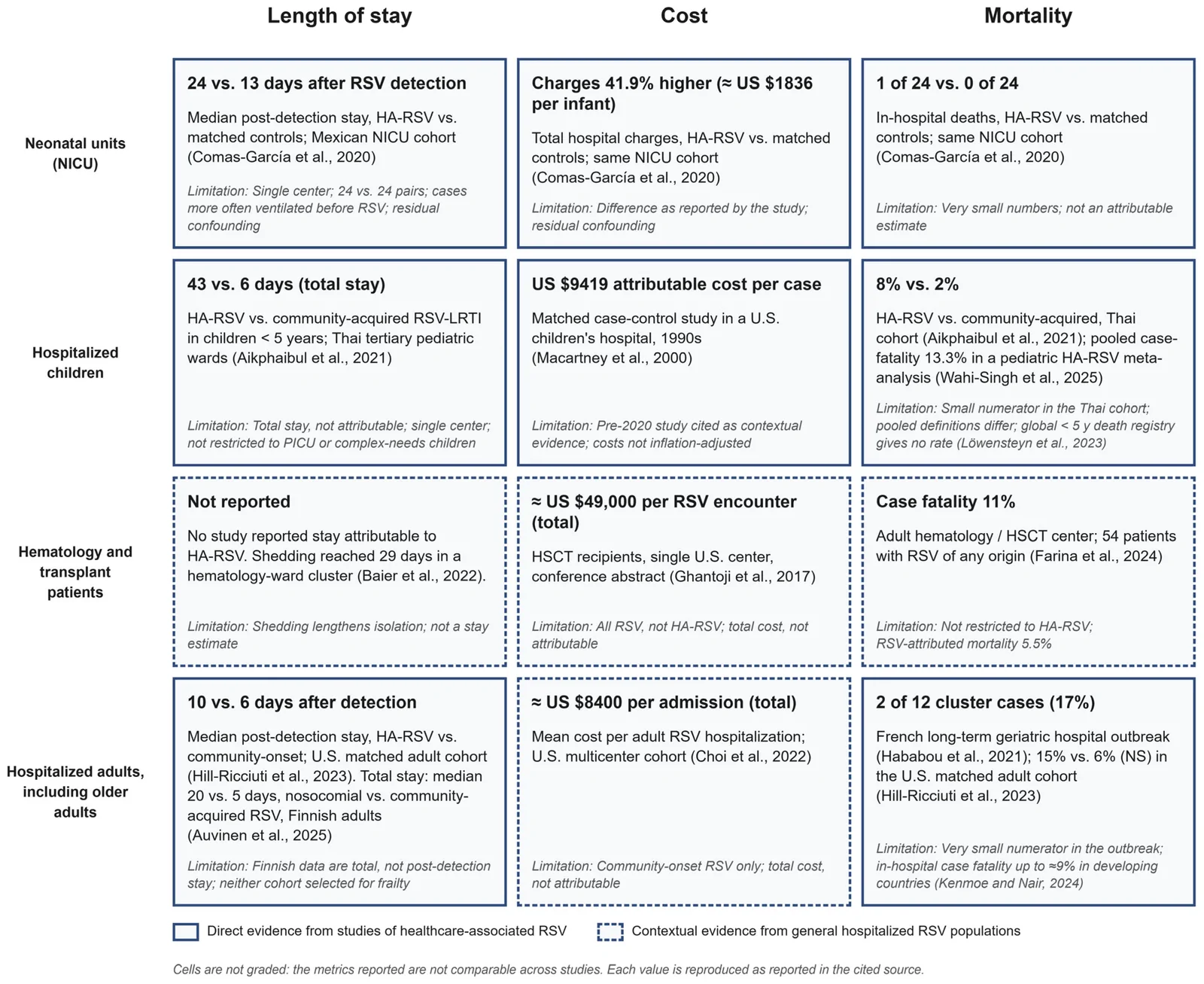
Evidence map of the reported burden of RSV in patient groups represented in the core studies. Rows are labeled according to the populations actually studied (neonatal units; hospitalized children; hematology and transplant patients; hospitalized adults, including older adults) rather than according to narrower risk groups, and each cell states the study population, the metric actually reported, its value, and its principal limitation. Differences between groups are reported as observed in the source studies and are not presented as attributable effects, because residual confounding cannot be excluded even in matched designs; cells are deliberately not graded, because the metrics are not comparable across studies. A solid border marks cells supported by direct evidence from studies of HA-RSV, and a dashed border marks cells supported only by contextual evidence from general hospitalized RSV populations; “not reported” indicates that no source in this review reported that domain for that group. Sources cited in the cells: [4,17,18,20,28,30,43,44,45,66,68,69,70]; the figure does not represent pooled quantitative estimates or a formal comparative analysis.

The smallest, sickest, and most immunologically fragile patients experience the most severe consequences. This stratified profile suggests that prevention should prioritize NICUs, PICUs, transplant wards, and geriatric units, while recognizing that lapses anywhere in the hospital can seed transmission to these high-risk zones. Figure 4 summarizes, for the patient groups actually studied, the burden domains for which evidence exists and indicates whether that evidence is specific to HA-RSV or contextual.

### 3.5. Contextual Evidence: Economic Burden of RSV Hospitalization in General Inpatient Populations

Excess costs associated with HA-RSV are described in Section 3.1.3; only two matched studies, both in pediatric settings, have quantified them (total hospital charges approximately US $1836 per infant, or 41.9%, higher than in matched controls in a Mexican NICU [4]; attributable cost US $9419 per nosocomial case in a U.S. children’s hospital [43]). The figures in this section refer to total RSV-related hospitalization costs in general inpatient populations, most of which are community-acquired; they indicate the resources consumed by RSV admissions but should not be read as the attributable burden of healthcare-associated infection.

**Hospitalization costs in adults.** In a systematic review of 42 economic studies, the national direct cost of RSV hospitalizations in the United States was estimated at US $1.5–4.0 billion per year for adults aged ≥60 years, with hospitalization accounting for most of the total and older age, comorbidities, and length of stay driving costs [71]. In a U.S. multicenter cohort (2017–2019), the mean cost per adult RSV hospitalization was approximately US $8400 [70]; across U.S. studies included in a systematic review, per-hospitalization estimates ranged from about US $8000 to more than US $50,000 (2022 US dollars), depending on population, methodology, and study period [71]. For immunocompromised hosts such as hematopoietic stem-cell transplant (HSCT) recipients, a single-center analysis reported in abstract form found an average total cost of approximately US $49,000 per RSV encounter, higher than for parainfluenza or influenza encounters in the same population [69].

**Cardiac complications.** Beyond respiratory disease, RSV admissions in older adults are frequently complicated by acute cardiac events—including acute heart failure and ischemic heart disease—which occurred in 22% of hospitalized adults aged ≥50 years in U.S. surveillance and were associated with a higher risk of ICU admission and in-hospital death [72]. These cardiac complications are rarely captured in infection-control analyses.

Cost estimates from different countries, healthcare systems, patient populations, and study periods are not directly comparable, so cross-country comparisons should be interpreted cautiously [71].

## 4. Discussion

### 4.1. Concurrent Circulation of RSV, Influenza, and SARS-CoV-2: Clinical Overlap and Resource Strain

A major post-pandemic challenge for hospitals is the concurrent circulation of RSV, influenza, and SARS-CoV-2—the “tripledemic”—which was observed from late 2022 to early 2023 [73]. Whether such overlap will recur regularly is uncertain (Section 3.2.1), but the 2022–2023 experience showed what concurrent circulation implies for hospital operations.

The syndromic overlap among these pathogens creates diagnostic blind spots: fever, cough, and dyspnea are clinically indistinguishable, so new-onset fever in any inpatient warrants differentiation among all three. Although co-infections are relatively uncommon, they may be associated with increased severity, including respiratory failure and prolonged hospitalization.

This convergence strains isolation capacity. CDC guidance for healthcare personnel entering the room of a patient with suspected or confirmed SARS-CoV-2 infection recommends Standard Precautions together with a NIOSH-approved N95 or higher-level respirator, gown, gloves, and eye protection, with a single-patient room preferred [59]; WHO permits a medical mask for routine care where local risk assessment supports it and reserves respirators for aerosol-generating procedures and other higher-risk situations [74]. Both reserve airborne infection isolation rooms for aerosol-generating procedures rather than routine care [58,59,74]; RSV requires contact precautions, and many institutions add droplet precautions, in a single room or cohort [58]. Concurrent outbreaks therefore compete for the same single rooms, cohort areas, and staff, rather than specifically for negative-pressure capacity, and can rapidly saturate isolation capacity and produce emergency-department (ED) overcrowding and bed-occupancy pressures that may facilitate nosocomial cross-transmission. Diagnostic speed thus becomes an operational priority, because rapid molecular testing can support timely patient-placement decisions: in a before–after study of emergency-department patients, rapid PCR for influenza and RSV shortened test turnaround, increased the proportion of patients receiving results before leaving the ED, and was associated with fewer hospital admissions, although ED length of stay did not differ significantly [75]. As contextual evidence of the resource use involved, RSV-positive encounters accounted for one-fifth of U.S. pediatric ICU days in 2017–2023 [65], and seasonal bronchiolitis surges can exhaust pediatric ICU capacity [76].

### 4.2. Integrated Prevention Strategies

The prevention of HA-RSV is best conceptualized using the “Swiss cheese” model of layered defenses originally described by Reason for accident causation in complex systems: each barrier has holes, but transmission occurs only when the holes in successive layers align, so that multiple imperfect layers together provide robust protection [77]. The model has been applied explicitly to hospital preparedness against respiratory viruses during the COVID-19 pandemic [78] and is used in this review to frame the measures below, which span hygiene, transmission-based precautions, environmental cleaning, diagnostics, workforce and visitor management, and immunoprophylaxis. For each measure, the principal source and level of supporting evidence is indicated—formal guidance (CDC, WHO, professional societies), observational outbreak evidence, or mechanistic/in vitro evidence—and Table 2 summarizes the measures by vulnerable population.

#### 4.2.1. Standard and Transmission-Based Precautions

Hand hygiene remains the cornerstone of prevention (formal guidance; supported by contact-transmission evidence in Section 3.3); alcohol-based rubs or soap and water before and after every patient or surface contact reduce fomite-mediated spread [54,55,58]. PPE is layered by risk: gloves and gowns for direct contact, with masks and eye protection when exposure to respiratory secretions is anticipated (formal guidance [58]). During aerosol-generating procedures, respiratory protection and room choice should follow institutional policy for the pathogens suspected, with enhanced protection where co-infection with an airborne-transmitted pathogen cannot be excluded (guidance-based; see Section 3.3) [56,58]. Patient placement follows a hierarchy—single rooms first, then cohorting of RSV-positive cases with dedicated staff, and, when isolation space is exhausted, spatial separation of at least 1 m (3 feet), the minimum given in CDC guidance for droplet precautions, with greater distances applied by many institutions (formal guidance; supported by outbreak evidence [6,17,58]). Negative-pressure (airborne infection isolation) rooms are not required for routine isolation of patients with RSV [58]. The importance of room configuration is supported by the 10-year Paris surveillance study, in which the incidence of healthcare-associated viral respiratory infection rose with the proportion of double-occupancy rooms on a ward [29]; where single rooms are unavailable, symptom-triggered bedside droplet precautions in multi-bed rooms were not inferior to single-room isolation for the combined influenza/RSV outcome in a Swiss pilot study, although RSV events were too few to judge RSV specifically (observational evidence [33,37]).

#### 4.2.2. Environmental Cleaning and Disinfection

RSV survives for hours to days on stainless steel and plastic, and for tens of minutes on hands, so high-touch surfaces can sustain transmission when cleaning lapses (experimental evidence [54,55]). RSV was efficiently inactivated in vitro by the tested alcohol-, aldehyde-, and hydrogen peroxide-based surface disinfectants [55]. Routine cleaning and disinfection of high-touch environmental surfaces should follow locally approved hospital protocols (formal guidance [58]).

#### 4.2.3. Early Detection and Diagnostics

Outbreak control starts with rapid case finding. Rapid molecular testing can markedly shorten turnaround time compared with standard laboratory PCR (2.4 h vs. 26.7 h in a before-and-after study in four EDs), enabling earlier isolation and clinical decision-making [75], whereas multiplex RT-PCR offers higher sensitivity and differentiation from influenza or SARS-CoV-2 for cohorting. During an outbreak, most IPC teams extend testing to roommates, exposed staff, and—where feasible—other symptomatic ward patients to define the perimeter (outbreak evidence [6,17,62]). Two core studies suggest a role for systematic screening beyond outbreaks: universal pre-admission RSV PCR screening of children during an early, unexpected epidemic was followed by an approximately 70% reduction in the proportion of nosocomial RSV infections in one Italian children’s hospital [34], and admission and weekly PCR prevalence screening on an adult hematology ward detected a monoclonal cluster while all four patients were still asymptomatic [18]; both are single-center observations, and the cost-effectiveness of screening outside high-risk units has not been established.

#### 4.2.4. Healthcare Worker Management

To limit transmission from mildly symptomatic staff, institutions should shift from passive screening to active structural support (observational evidence on presenteeism [5,60]). Non-punitive sick-leave policies de-stigmatize absenteeism and reduce the economic pressure driving presenteeism. Healthcare workers who meet age- or risk-based national eligibility criteria should be offered RSV vaccination [1,79]. However, universal RSV vaccination is not currently recommended for the healthcare workforce on occupational grounds; therefore, symptom reporting, non-punitive sick leave, masking, and adherence to transmission-based precautions remain the principal occupational-health measures (formal guidance [58,79]).

#### 4.2.5. Visitor Policies

Screening visitors for symptoms, enforcing hand hygiene and masking, and restricting non-essential visits in high-risk units curb external seeding (outbreak evidence: visitor-associated introductions were documented in the NICU outbreak investigated by whole-genome sequencing [6]); clear signage and compassionate communication secure compliance without alienating families.

#### 4.2.6. Prophylaxis and Immunization: An Indirect Layer

The products discussed here were developed and licensed to prevent RSV disease in individuals; none of the currently recommended products (nirsevimab, clesrovimab, the maternal vaccine, and the adult vaccines) was evaluated for preventing healthcare-associated acquisition or nosocomial outbreaks in the studies retrieved for this review, although palivizumab has been used as an adjunct during a neonatal outbreak [16], and the multicenter pediatric cohort explicitly identified their impact on HA-RSV as unknown [31]. Their relevance to hospital infection prevention is therefore indirect and, at present, hypothetical: fewer and less severe RSV admissions may reduce the number of infectious inpatients and the opportunities for in-hospital transmission, and protecting eligible long-stay patients and healthcare workers may reduce the susceptible pool, but neither effect has been measured. The following summarizes current guidance (formal recommendations, re-checked on 16 September 2026); recommendations differ between jurisdictions and are subject to change.

**Infants.** Long-acting monoclonal antibodies, nirsevimab and clesrovimab, where approved and available, are recommended in several jurisdictions for infants younger than 8 months born during or entering their first RSV season who are not protected by maternal vaccination, with nirsevimab also recommended for high-risk children aged 8–19 months entering their second season; in the United States, administration before discharge from the birth hospital is the recommended practice for infants born during the season [80,81,82,83]. Administration primarily protects the infant after discharge, including against readmission; in a pragmatic, randomized trial, nirsevimab reduced RSV-associated hospitalization by 83% [84]. Guidance on administration during the admission itself has changed: the 2023 ACIP recommendation stated that nirsevimab was not recommended for the prevention of hospital-acquired RSV infection, whereas current CDC clinical guidance states that, for eligible infants with prolonged hospitalization shortly before or during the RSV season, providers may consider administering RSV antibody during the hospitalization to prevent healthcare-associated RSV disease, on the basis of clinical judgment [80,82]. This recommendation focuses on protecting the individual hospitalized infant against RSV disease; it is not evidence that immunoprophylaxis interrupts nosocomial transmission, which has not been evaluated. Product availability and eligibility criteria vary across jurisdictions [81,83].

**Maternal.** The bivalent maternal RSV prefusion-F vaccine has shown efficacy of approximately 57–82% against medically attended and severe RSV lower-respiratory illness in infants during the first months of life, depending on endpoint and follow-up interval [85], and is recommended at 32–36 weeks’ gestation in the United States, seasonally in temperate settings [86]. WHO recommends a single dose from 28 weeks’ gestation onwards, with no upper gestational-age limit other than active labor [83]; the two schedules are not interchangeable, and the applicable national recommendation should be followed. Maternal vaccination provides transplacental antibodies that protect the infant from birth, but there is no direct evidence that it reduces healthcare-associated acquisition during the birth hospitalization.

**Older adults.** RSV vaccines for older adults have demonstrated substantial protection against RSV-associated lower-respiratory disease and hospitalization. In the United States, a single dose is currently recommended for all adults aged ≥75 years and for adults aged 50–74 years who are at increased risk of severe RSV disease [1,79]. Three vaccines (the adjuvanted RSVPreF3 vaccine, the bivalent prefusion F vaccine, and the mRNA-1345 vaccine) are in addition licensed by the FDA for adults aged 18–49 years at increased risk of RSV-associated lower respiratory tract disease, the most recent of these extensions having been approved in March 2026 [79,87]. Licensure should be distinguished from vaccination recommendations: ACIP has not yet issued a recommendation for this age group [79]. This age band includes many hematology and transplant patients. Vaccination of eligible long-stay inpatients and healthcare workers who meet age- or risk-based criteria may reduce the susceptible pool in hospitals, but this effect has not been measured.

**Note on timing.** Align RSV immunization timing with local surveillance, regional seasonality, product-specific recommendations, and national guidance to ensure protection is established before RSV activity increases.

### 4.3. Impact of COVID-19 Control Measures on RSV Prevention

Universal masking, visitor restrictions, and intensified disinfection during 2020–2021 coincided with a near-disappearance of RSV, including in hospitals, providing indirect real-world evidence that layered NPIs can markedly reduce hospital RSV transmission (systematic review and surveillance evidence [9,46,48]). The core studies add direct hospital-level observations: nosocomial RSV disappeared after universal masking in a Hong Kong hospital network [32], fell by 70% at a U.S. cancer center [36], and combined with influenza, fell by 53% in a U.S. academic hospital even after adjustment for community activity [35]; by contrast, the HA-RSV transmission index in six U.S. children’s hospitals did not decrease during universal masking in 2020–2021 [24], and RSV-specific effects could not be shown in an Australian children’s hospital because of low counts [38]. These observational comparisons cannot separate the contribution of hospital measures from the collapse of community RSV circulation during the same period. However, such resource-intensive NPIs are hard to sustain; adherence slipped once mandates expired and RSV resurged. Hospitals therefore need to balance surge-triggered re-escalation against staff fatigue and supply constraints, favoring agile, risk-based IPC protocols [7,11,49].

### 4.4. Challenges and Limitations in Management

Despite the availability of integrated prevention strategies, implementation is frequently hampered by diagnostic, behavioral, and logistical barriers.

#### 4.4.1. Diagnostic Hurdles

Accurate, timely diagnosis is the first hurdle. RSV is shed for days by asymptomatic or mildly symptomatic carriers, roughly one in five infected staff in an early outbreak investigation [2], and by immunosuppressed patients, obscuring the outbreak perimeter [3]. Atypical presentation in the frail elderly or extremely preterm infants leads to late or no testing, and even when suspicion is high, central-laboratory PCR can exceed 24 h at peak season, delaying isolation [75]. Under-recognition is compounded by incomplete documentation: a virus-specific diagnosis code was recorded for only 17–18% of adult HA-RSV cases in two cohorts [28,30] and for 30% of pediatric cases [24], and none of 13 HA-RSV deaths in one adult cohort had RSV on the death certificate [30]. Co-circulating influenza A and SARS-CoV-2 further cloud interpretation through overlapping clinical presentations.

#### 4.4.2. Behavioral Fatigue and Workforce Issues

Maintaining high-fidelity prevention behaviors is equally challenging. Qualitative interviews conducted among ICU healthcare workers early in the pandemic identified individual, managerial, and organizational barriers to hand-hygiene compliance, including workload and resource constraints [88]. The workforce crisis compounds this: staff shortages force nurses to be cohorted across multiple rooms, increasing cross-transmission risk, and a longitudinal study in a regional pediatric hospital reported a peak in respiratory infection-related sick leave among HCWs during 2020–2021, followed by a decline in 2022–2023 [89].

Presenteeism, working while ill, is a recurring theme, but the available estimates measure different quantities and should not be conflated. In a prospective cohort of 2505 healthcare personnel in two Israeli hospitals, staff continued to work during 92% of acute respiratory illness episodes of any cause (RSV illness incidence was 2.5 per 100 person-seasons); this figure describes the proportion of illness episodes worked through and is not specific to RSV [5]. In a U.S. surveillance cohort, 46% of on-duty HCWs reported having worked while ill [60]. By contrast, outbreak investigations in neonatal and pediatric units report the proportion of ward staff who became infected during an outbreak—56% in one early report, of whom some continued to work while mildly symptomatic [2,3]. The two sets of figures thus describe, respectively, how often ill staff keep working and how many staff become infected during an outbreak, and are not comparable estimates of the same quantity. Presenteeism is driven by pressure to support understaffed teams, the mild presentation of adult RSV, and the absence of paid sick leave; a mildly symptomatic staff member may thus become a source of transmission within a NICU or transplant ward [5]. Visitor policies can also erode under family pressure, exposing gaps in these defensive layers.

#### 4.4.3. Logistical and Financial Constraints

Logistical constraints add further complexity. The relevant constraint for RSV is the availability of single-patient rooms and the feasibility of cohorting with dedicated staff during surges; because RSV is transmitted by contact and short-range respiratory particles, standard single rooms are sufficient, and negative-pressure rooms are not required for routine isolation [58]; their use during aerosol-generating procedures depends on the pathogens suspected, the possibility of co-infection with an airborne-transmitted pathogen, and institutional policy rather than on an RSV-specific requirement. Many hospitals nevertheless lack sufficient single rooms during surges, forcing reliance on spatial distancing in open bays—an imperfect compromise [17,28]. Seasonal bronchiolitis surges can likewise strain pediatric ICU capacity, requiring strategies that minimize ICU stay and preserve beds for other critically ill patients [76]. The cost of PPE, rapid molecular tests, and overtime labor is substantial, particularly for resource-limited institutions. Distinguishing community-acquired from healthcare-associated infection is also complicated by uncertainty in exposure timing, variable symptom onset, and differences in surveillance definitions; estimates differ two- to three-fold according to the definition and healthcare exposures included [25,40]; genomic epidemiology offers a solution but remains resource-intensive and rarely available in real time [6,18,62].

#### 4.4.4. Environmental and Immunization Gaps

Finally, vulnerabilities persist in the physical environment and immunization landscape. Because RSV remains viable on surfaces for hours, lapses in disinfection can sustain transmission despite good hand hygiene [54,55]. Unlike influenza, there is no occupational recommendation for HCW RSV vaccination; adult RSV vaccines are recommended for age- and risk-defined adult groups but are not recommended for the healthcare workforce on occupational grounds [1,79]. The extent to which infant immunoprophylaxis and adult vaccination will reduce HA-RSV has not been measured and is an important research gap [31]. As hospitals confront overlapping waves, infection-prevention programs must manage three distinct testing algorithms and PPE policies—an operational burden that amplifies every gap in training, staffing, and supply chains.

### 4.5. Limitations of the Current Evidence and of This Review

The literature on HA-RSV is largely composed of single-center outbreak reports and retrospective series with inconsistent case definitions, limiting causal inference; few studies quantify the incremental benefit of individual bundle components or address adults in low- and middle-income countries. Genomic epidemiology is not yet standard, and economic analyses often overlook indirect costs such as staff absenteeism. Although 29 primary studies published since 2020 reported HA-RSV cases, most were single-center and retrospective; case numbers ranged from 1 to 136, and only three compared RSV-specific outcomes against a matched comparison group; the attributable burden of HA-RSV therefore rests on a modest evidence base, and several widely cited figures (secondary attack rates, staff infection rates, asymptomatic shedding) derive from outbreak investigations conducted decades ago.

This review has its own limitations. Although a structured search with independent dual screening was used to limit selection bias, the review remains narrative: formal risk-of-bias assessment and quantitative synthesis were not performed, study quality was considered descriptively (Section 2.3), and contextual sources were included at the authors’ discretion, so that selection bias cannot be excluded. The database search was limited to PubMed/MEDLINE and Europe PMC; although Europe PMC extends coverage beyond MEDLINE to PubMed Central and preprint servers, studies indexed only in other databases (for example, Embase, Scopus, or Web of Science) may have been missed, a limitation mitigated only in part by hand-searching of reference lists. Nine reports that passed title/abstract screening (four from the original search and five from the update search) could not be retrieved in full text and were therefore not assessed for eligibility; two of the four from the original search reported healthcare-associated infections in a form that could not be resolved from their abstracts—nosocomial infections among children hospitalized with RSV, and pooled healthcare-associated respiratory viral infections—so a small number of eligible HA-RSV cases may have been missed. Restricting the core evidence set to studies published from 2020 onward, chosen as a pragmatic boundary reflecting the pandemic transition and recent practice, excludes older primary studies from the core set, although the most influential of these are cited as contextual evidence. Definitions of HA-RSV varied across studies and were not harmonized. Finally, guidance on RSV immunization and isolation precautions is evolving and differs between jurisdictions; the recommendations cited reflect the versions available on 16 September 2026, and readers should consult current national guidance. The update of Search 1 covered records up to 21 September 2026, but was restricted to English-language reports, so non-English reports from that period may have been missed; Search 2 (post-pandemic epidemiology) was not updated beyond 31 October 2025.

Priorities for future research include the evaluation of infant immunoprophylaxis and adult vaccination as tools for preventing HA-RSV; clarification of the contribution of smaller airborne particles to transmission in clinical settings; the cost-effectiveness of screening asymptomatic carriers and of individual infection-control measures; standardized surveillance definitions that allow incidence to be compared across hospitals; and studies of HA-RSV in adults and in low- and middle-income countries.

## 5. Conclusions

Healthcare-associated RSV is under-recognized and is associated with prolonged hospitalization, escalation of respiratory support, excess cost and, in children, higher mortality, with the greatest burden borne by preterm neonates, children with complex medical needs, transplant recipients, and frail older adults. Post-pandemic epidemiology has become less predictable: RSV activity has occurred earlier and out of season, concurrent circulation with influenza and SARS-CoV-2 has been observed, and calendar-based preparedness is no longer sufficient, so that infection-prevention programs should be escalated according to local surveillance. The primary layers of prevention remain hand hygiene, contact and droplet precautions with appropriate patient placement, environmental disinfection, rapid molecular diagnostics, and supportive policies for healthcare workers and visitors, applied together so that lapses in one layer are covered by another. Long-acting monoclonal antibodies and maternal and older-adult vaccines are recommended for eligible populations because they reduce severe RSV disease; their contribution to hospital infection prevention is indirect, and whether they reduce healthcare-associated transmission remains to be demonstrated.

## Figures and Tables

**Table 2:** Key measures for preventing healthcare-associated RSV transmission in vulnerable inpatient populations, with the principal source and level of supporting evidence.

Population/Unit	Core Measures (All Units)	Population-Specific Considerations	Immunoprophylaxis and Vaccination (Indirect Layer)	Principal Source and Level of Evidence
All units	Hand hygiene before and after every patient and surface contact; contact precautions with droplet precautions added per local policy (gloves, gown, mask, eye protection when secretions anticipated); single room, or cohorting of RSV-positive patients with dedicated staff, then spatial separation (≥1 m/3 feet minimum per CDC droplet-precaution guidance; many institutions use 2 m) if isolation space is exhausted; routine disinfection of high-touch surfaces; rapid molecular testing of symptomatic inpatients and, in outbreaks, of roommates and exposed staff; HCW symptom reporting and non-punitive sick leave; visitor symptom screening, masking and restriction in outbreaks; surveillance-triggered escalation rather than calendar-based activation	AIIRs not required for routine RSV care; during AGPs, respiratory protection and room choice per institutional policy for the pathogens suspected (no RSV-specific requirement)	Offer age- or risk-based RSV vaccination to eligible HCWs and long-stay patients; no occupational recommendation exists	G: CDC isolation-precaution guideline [58]; O: outbreak investigations and cohorts [6,17,28]; O: universal masking and pandemic-era measures [24,32,35,36]; O: room occupancy and bedside droplet precautions [29,33,37]; M: contact/fomite and disinfectant studies [54,55]; G: adult vaccine guidance [1,79]
Preterm neonates and NICU	As above	Prompt isolation or cohorting of the first case (secondary attack rates up to 45% when delayed); daily HCW symptom attestation; restriction of child visitors and visitors with respiratory symptoms; expanded testing of asymptomatic exposed infants during outbreaks; consider WGS to define introductions	Nirsevimab (or clesrovimab where available) before discharge for infants born during or entering their first RSV season who are not protected by maternal vaccination—the purpose is protection after discharge; for eligible infants with prolonged hospitalization shortly before or during the season, CDC guidance allows administration during the admission to prevent healthcare-associated RSV disease in that infant, although prevention of nosocomial transmission has not been demonstrated; maternal RSVpreF at 32–36 weeks’ gestation (U.S.) or from 28 weeks’ gestation (WHO)	O: NICU outbreaks with molecular typing [6,15,16]; O: NICU cohort (LOS, cost) [4]; O: early outbreak reports [2,3]; G/T: ACIP and WHO recommendations, randomized trials [80,81,82,83,84,85,86]
Children with complex medical needs and PICU	As above	Low threshold for testing new respiratory deterioration ≥72 h after admission; consider pre-admission RSV PCR screening during epidemics; cohorting of RSV-positive children away from those with cardiopulmonary or neuromuscular disease; surge plans to preserve ICU capacity	Nirsevimab for high-risk children aged 8–19 months entering their second season (where recommended); age-appropriate immunization status checked on admission	O: multicenter HA-RSV surveillance and cohort [24,31]; O: Thai HA-RSV cohort [20]; O: PICU surveillance [21]; O: pre-admission screening [34]; O: surveillance definitions [25]; G: ACIP [80]; contextual: PICU utilization [65,76]
HSCT and SOT recipients	As above	Extended isolation for the duration of shedding (may exceed 3 weeks); admission and periodic PCR screening during community activity; testing of staff and roommates when ≥2 cases occur; restriction of visitors with any respiratory symptoms; consider WGS for cluster investigation	No product is licensed specifically for transplant recipients, but recipients who meet age- or risk-based criteria are eligible for adult RSV vaccination (U.S.: all adults ≥75 years, and adults 50–74 years with moderate or severe immune compromise). Three vaccines are in addition FDA-licensed for adults 18–49 years at increased risk, an age band that includes many transplant recipients, although ACIP has not yet issued a recommendation for that group. Vaccination of eligible household contacts and HCWs per age/risk criteria	O: hematology-ward cluster detected by screening [18]; O: HSCT-unit outbreak with WGS [62]; O: cancer-center surveillance [36]; O: prolonged shedding [3]; contextual: hematology cohort outcomes [66]; G: ACIP/CDC adult vaccine guidance [1,79]
Frail older adults and geriatric units	As above	Atypical presentation (no fever) warrants testing new respiratory or cardiac decompensation; room confinement and suspension of communal activities during outbreaks; masking of roommates when single rooms are unavailable; attention to under-diagnosis and coding	Single-dose RSV vaccine for all adults ≥75 years and adults 50–74 years at increased risk (U.S. guidance); consider vaccination of eligible long-stay patients	O: geriatric outbreak with genotyping [17]; O: adult cohorts and surveillance [26,28,30,40]; G: ACIP/CDC adult guidance [1,79]; contextual: RSV-NET surveillance [67]

RSV, respiratory syncytial virus; HA-RSV, healthcare-associated RSV; NICU, neonatal intensive care unit; PICU, pediatric intensive care unit; HSCT, hematopoietic stem-cell transplant; SOT, solid-organ transplant; HCW, healthcare worker; PPE, personal protective equipment; AGP, aerosol-generating procedure; AIIR, airborne infection isolation room; PCR, polymerase chain reaction. Levels of evidence: G, formal guidance (CDC, WHO, professional societies); O, observational evidence from outbreak investigations or cohort studies of HA-RSV; M, mechanistic or in vitro evidence; T, randomized trials of individual protection against RSV disease (not of prevention of nosocomial transmission). Core measures apply to all units and are listed once; population-specific rows list additional considerations.

## Data Availability

No new data were created or analyzed in this study. All data discussed are available in the cited published sources. The complete search strategies (Table S1) are provided as Supplementary Materials.

## Supplementary Materials

The following supporting information can be downloaded at: https://www.mdpi.com/article/10.3390/healthcare14193335/s1; Table S1, Complete database-specific search strategies.

## Abbreviations

The following abbreviations are used in this manuscript:

RSV	Respiratory syncytial virus
IPC	Infection prevention and control
NPI	Non-pharmaceutical intervention
PPE	Personal protective equipment
HCW	Healthcare worker
NICU	Neonatal intensive care unit
PICU	Pediatric intensive care unit
ICU	Intensive care unit
HSCT	Hematopoietic stem-cell transplant
PCR	Polymerase chain reaction
RT-PCR	Reverse-transcription polymerase chain reaction
ED	Emergency department
COPD	Chronic obstructive pulmonary disease
CDC	Centers for Disease Control and Prevention
WHO	World Health Organization
HA-RSV	Healthcare-associated respiratory syncytial virus
LRTI	Lower respiratory tract infection
RTI	Respiratory tract infection
ILI	Influenza-like illness
WGS	Whole-genome sequencing
AGP	Aerosol-generating procedure
AIIR	Airborne infection isolation room
SOT	Solid-organ transplant
LOS	Length of stay
ACIP	Advisory Committee on Immunization Practices
OR	Odds ratio
CI	Confidence interval
