# Supplementary material for: Nosocomial Respiratory Syncytial Virus in the Post-Pandemic Era: Evolving Epidemiology and Integrated Prevention Strategies
Source: Healthcare (Basel). 2026 Oct 6;14(19):3335. doi: 10.3390/healthcare14193335 (PMC13665019; doi:10.3390/healthcare14193335)
Supplement: Supplementary file 1 [file healthcare-14-03335-s001.zip › healthcare-4578903-supplementary.pdf]

Supplementary Materials

Table S1. Complete database-specific search strategies. Two complementary searches were run: Search 1 targeted healthcare-associated RSV and deliberately contained no pandemic-related terms, so that pre-pandemic evidence on transmission and prevention was captured; Search 2 targeted post-pandemic epidemiology. All searches were limited to human studies by the eligibility criteria applied at screening; no language limits were applied. Records from both databases were deduplicated before screening (see Figure 1 of the main text).

Database/Source	Date Searched	Search String/Strategy	Records Retrieved (n)
**Search 1 — Healthcare-associated RSV (no pandemic-related terms); 1 January 2000–31 October 2025**			
PubMed/MEDLINE	16 September 2026	("Respiratory Syncytial Virus, Human"[Mesh] OR "Respiratory Syncytial Virus Infections"[Mesh] OR "respiratory syncytial virus"[tiab] OR RSV[tiab]) AND ("Cross Infection"[Mesh] OR nosocomial[tiab] OR "hospital-acquired"[tiab] OR "hospital acquired"[tiab] OR "healthcare-associated"[tiab] OR "healthcare associated"[tiab] OR "health care-associated"[tiab] OR "hospital outbreak"[tiab] OR "hospital-onset"[tiab]) AND 2000/01/01:2025/10/31[dp]	391
Europe PMC (REST API, all sources)	17 September 2026	(TITLE:"respiratory syncytial virus" OR ABSTRACT:"respiratory syncytial virus" OR KW:"respiratory syncytial virus" OR TITLE:RSV OR ABSTRACT:RSV OR KW:RSV) AND (TITLE:nosocomial OR ABSTRACT:nosocomial OR KW:nosocomial OR TITLE:"hospital-acquired" OR ABSTRACT:"hospital-acquired" OR KW:"hospital-acquired" OR TITLE:"hospital acquired" OR ABSTRACT:"hospital acquired" OR KW:"hospital acquired" OR TITLE:"healthcare-associated" OR ABSTRACT:"healthcare-associated" OR KW:"healthcare-associated" OR TITLE:"healthcare associated" OR ABSTRACT:"healthcare associated" OR KW:"healthcare associated" OR TITLE:"health care-associated" OR ABSTRACT:"health care-associated" OR KW:"health care-associated" OR TITLE:"hospital outbreak" OR ABSTRACT:"hospital outbreak" OR KW:"hospital outbreak" OR TITLE:"hospital-onset" OR ABSTRACT:"hospital-onset" OR KW:"hospital-onset" OR TITLE:"cross infection" OR ABSTRACT:"cross infection" OR KW:"cross infection") AND FIRST_PDATE:[2000-01-01 TO 2025-10-31]	431
**Search 2 — Post-pandemic RSV epidemiology in hospital settings; 1 January 2020 – 31 October 2025**			
PubMed/MEDLINE	16 September 2026	("Respiratory Syncytial Virus, Human"[Mesh] OR "Respiratory Syncytial Virus Infections"[Mesh] OR "respiratory syncytial virus"[tiab] OR RSV[tiab]) AND ("COVID-19"[Mesh] OR "COVID-19"[tiab] OR "SARS-CoV-2"[tiab] OR pandemic[tiab] OR "post-pandemic"[tiab] OR "non-pharmaceutical interventions"[tiab]) AND (seasonality[tiab] OR seasonal[tiab] OR resurgence[tiab] OR "immunity debt"[tiab] OR "off-season"[tiab] OR interseasonal[tiab] OR "out-of-season"[tiab] OR "before and during"[tiab]) AND (hospital[tiab] OR hospitals[tiab] OR hospitalized[tiab] OR hospitalised[tiab] OR hospitalization[tiab] OR hospitalisation[tiab] OR hospitalizations[tiab] OR inpatient[tiab] OR inpatients[tiab] OR "Hospitalization"[Mesh]) AND 2020/01/01:2025/10/31[dp]	394
Europe PMC (REST API, all sources)	17 September 2026	(TITLE:"respiratory syncytial virus" OR ABSTRACT:"respiratory syncytial virus" OR KW:"respiratory syncytial virus" OR TITLE:RSV OR ABSTRACT:RSV OR KW:RSV) AND (TITLE:"COVID-19" OR ABSTRACT:"COVID-19" OR KW:"COVID-19" OR TITLE:"SARS-CoV-2" OR ABSTRACT:"SARS-CoV-2" OR KW:"SARS-CoV-2" OR TITLE:pandemic OR ABSTRACT:pandemic OR KW:pandemic OR TITLE:"post-pandemic" OR ABSTRACT:"post-pandemic" OR KW:"post-pandemic" OR TITLE:"non-pharmaceutical interventions" OR ABSTRACT:"non-pharmaceutical interventions" OR KW:"non-pharmaceutical interventions") AND (TITLE:seasonality OR ABSTRACT:seasonality OR KW:seasonality OR TITLE:seasonal OR ABSTRACT:seasonal OR KW:seasonal OR TITLE:resurgence OR ABSTRACT:resurgence OR KW:resurgence OR TITLE:"immunity	487

		debt" OR ABSTRACT:"immunity debt" OR KW:"immunity debt" OR TITLE:"off-season" OR ABSTRACT:"off-season" OR KW:"off-season" OR TITLE:interseasonal OR ABSTRACT:interseasonal OR KW:interseasonal OR TITLE:"out-of-season" OR ABSTRACT:"out-of-season" OR KW:"out-of-season" OR TITLE:"before and during" OR ABSTRACT:"before and during" OR KW:"before and during") AND (TITLE:hospital* OR ABSTRACT:hospital* OR KW:hospital* OR TITLE:inpatient* OR ABSTRACT:inpatient* OR KW:inpatient*) AND FIRST_PDATE:[2020-01-01 TO 2025-10-31]	
**Other sources**			
Hand-searching of reference lists of included articles and pertinent reviews; guidance websites (WHO; U.S. CDC/ACIP; infection-control societies)	Throughout the review; guidance websites re-checked 16 September 2026	Reference lists screened manually; guidance documents and position papers on RSV immunization and isolation precautions. Includes the CDC RSV immunization guidance page for infants and young children and the FDA product page for the adjuvanted RSVPreF3 vaccine, both added during revision. Sources not already retrieved by Searches 1–2 were added.	48 (sources retained and cited)
**Total**			
Records identified from databases / after deduplication		Deduplication across searches and databases by PMID, DOI and title; preprints of articles already retrieved in published form were treated as duplicates.	1703 / 868

Notes. [tiab], title/abstract field (PubMed); [Mesh], Medical Subject Headings; [dp], publication date. In Europe PMC, TITLE, ABSTRACT and KW denote the title, abstract and keyword fields, and FIRST_PDATE the first publication date; Europe PMC indexes MEDLINE together with PubMed Central, preprint servers and other sources, so that most of its records duplicated PubMed records and were removed before screening. Record counts are those returned on the dates shown; counts for Searches 1 and 2 were summed before deduplication. Conference abstracts and preprints were screened but were not eligible for the core evidence set. RSV, respiratory syncytial virus; WHO, World Health Organization; CDC, Centers for Disease Control and Prevention; ACIP, Advisory Committee on Immunization Practices.

Update of Search 1 (run 21 September 2026). The Search 1 strings above were re-run in PubMed/MEDLINE with the date limit ("2025/11/01"[dp] : "2026/09/21"[dp]) and in Europe PMC with FIRST_PDATE:[2025-11-01 TO 2026-09-21]; no language filter was applied in the database queries, and the restriction of the update to English-language reports was applied at title/abstract screening (one Spanish-language record was excluded on this basis). PubMed/MEDLINE returned 21 records and Europe PMC 22 records (19 duplicates of PubMed records; 3 unique: 1 preprint and 2 conference abstracts), giving 24 unique records; the original 2000–2025 PubMed string reproduced the original 391 records on the same date. Screening outcomes are shown in Figure 1.
